# Updated chorology, habitat ecology, conservation assessment and habitat suitability of *Centaurea jankae* in Romania and Bulgaria

**DOI:** 10.3389/fpls.2026.1904573

**Published:** 2026-09-18

**Authors:** Simona Dumitriţa Chirilă, Nicu Alexandru Gîlea, Mihai Doroftei, Stoyan Nikolov, Kiril Vassilev

**Affiliations:** 1 Danube Delta National Institute for Research and Development, Tulcea, Romania; 2 Doctoral School Simion Mehedinți, Faculty of Geography, University of Bucharest, Bucharest, Romania; 3 Institute of Biodiversity and Ecosystem Research, Bulgarian Academy of Sciences, Sofia, Bulgaria

**Keywords:** calcareous steppe grasslands, *Centaurea jankae*, conservation assessment, Dobrogea, habitat fragmentation, Maxent

## Abstract

*Centaurea jankae* is a rare species with a restricted distribution in southeastern Romania and northeastern Bulgaria. This study aimed to update the chorology of *C*. *jankae*, characterise its habitat ecology and population-level variation, assess its local conservation condition, and model its current potential distribution and habitat suitability. Soil samples and data on topographic and bioclimatic factors were collected to assess environmental conditions. To update the species’ chorology, locations reported in the literature were verified in the field. The current potential distribution of the species was modelled in MaxEnt based on 27 presence records and five environmental variables. Of the 14 populations previously reported, six were confirmed: three locations in Romania (Jurilovca–Capul Doloşman, Palazu Mic, and Slava Rusă) and three in Bulgaria (Nevsha, Ravna, and Venchan). Recorded abundance ranged from 1,199 to 4,589 individuals among populations, while flowering individuals represented 9.3–47.1% of the recorded individuals. Ecologically, *C. jankae* was associated with xerothermal conditions and predominantly sunny aspects and occurred across a broad range of slopes, with higher cover tending to occur on moderately inclined slopes. The species occurred on alkaline soils showing substantial among-population variation in nutrient status. Populations in Romania are linked to drier conditions and more fertile soils, while populations in Bulgaria occur under wetter, more climatically stable conditions. The MaxEnt model yielded a mean test AUC of 0.930 (± 0.056) across cross-validation replicates. Land use/land cover showed the highest percent contribution (42.8%) and permutation importance (39.4%) in the fitted model, while soil type accounted for 24.3% of percent contribution. The potential habitat of *C. jankae* covered 3,562.68 km^2^ (11.2%) of the study area, of which 1,025.31 km^2^ (3.2%) were identified as highly suitable habitat. Local conservation condition was assessed as unfavourable–inadequate (U1) for four populations and unknown (XX) for two populations. In conclusion, the species’ restricted distribution, habitat fragmentation, and anthropogenic pressures highlight its vulnerability and the need to implement appropriate conservation measures to maintain *C. jankae* populations in the long term.

## 1 Introduction

One of the most significant global environmental issues is biodiversity loss (UNEP, 2021; Yang et al., 2025), with a substantial number of plant species facing extinction (Goettsch et al., 2015). Grassland ecosystems are among the most widespread terrestrial ecosystems globally (Liu et al., 2024), and cover approximately 30–40% of the Earth’s land surface (Balasubramanian et al., 2020) and 69% of the Earth’s agricultural land area (Bardgett et al., 2021). However, they are affected by environmental changes (land degradation, biodiversity loss, and decline in ecosystem services) and human activities (agricultural expansion, industrial development, and overgrazing; Guo et al., 2023; Liu et al., 2024). In the context of biodiversity decline, the conservation of rare plant species is a current priority (Xu and Zang, 2023). Anthropogenic and natural factors disproportionately affect species with strict environmental requirements and restricted distributions. Forest-steppe ecosystems may harbour numerous endemic and relict plant species, while ongoing habitat degradation may threaten their persistence (Shumilovskikh et al., 2021).

Among the plant species of high conservation value is *Centaurea jankae* D. Brândză, a member of the family Asteraceae (Compositae) (Greuter and von Raab-Straube, 2006). From a systematic perspective, *C. jankae* belongs to *Centaurea* sect. *Acrocentron* (Cass.) DC., based on morphological and molecular evidence (Font et al., 2009; Novaković et al., 2022), although it was previously assigned to sect. *Hyaleoloma* Dostál (Dostál, 1976). *Centaurea jankae* is considered a Dobrogean paleoendemic taxon, possibly originating near the end of the Tertiary (Petrova, 2007). Its current distribution is highly fragmented and has been interpreted as the remnant of a formerly wider range.

*Centaurea jankae* is endemic to Dobrogea (southeastern Romania and northeastern Bulgaria), having a restricted distribution area and fragmented populations (Bilz, 2011). The species is listed in Annexes II and IV of the Habitats Directive 92/43/EEC (Council of the European Communities, 1992), requiring the designation of Special Areas of Conservation and strict protection. It is also included in Appendix I of the Bern Convention (1979). Globally, the species is assessed as vulnerable (VU) on the IUCN Red List and is recognised as a taxon of high conservation concern (Bilz, 2011). In the Romanian literature (Boşcaiu et al., 1994; Dihoru and Dihoru, 1994; Oltean et al., 1994; Ciocârlan, 1999; Negrean and Anastasiu, 2003), it has been variously described as rare, endangered or threatened.

*Centaurea jankae* occurs on sunny, calcareous slopes at low elevations and is characteristic of Ponto-Sarmatian xerophilous steppe grasslands (Petrova, 2007). Its preferred habitats are natural grasslands, although the species may also occur sporadically on cultivated land or in sparsely vegetated areas when edaphic and climatic conditions are favourable (EUNIS species factsheet, 2025). The floristic composition is dominated by plant species such as *Stipa pulcherrima* K. Koch, *S. capillata* L., *Festuca valesiaca* Gaudin, *Chrysopogon gryllus* (L.) Trin., *Bothriochloa ischaemum* (L.) Keng, *Carex humilis* Leyss., and *Satureja coerulea* Janka, as well as shrubs such as *Paliurus spina*-*christi* Mill (Petrova, 2007; Bilz, 2011).

Due to habitat fragmentation, small population sizes and anthropogenic pressures, populations are particularly vulnerable to further decline (Bilz, 2011). The main reported or potential threats include overgrazing, climate change (droughts, precipitation deficits), the establishment and spread of invasive species, the abandonment of traditional grazing (which favours the establishment of shrubs), and human activities such as tourism-related disturbance and waste disposal. The conservation of this species requires regular population monitoring, appropriate grazing management, the maintenance of open habitats by limiting woody vegetation encroachment, the control of invasive species and the removal of illegally deposited waste. Given its restricted distribution and its inclusion in Annexes II and IV of the Habitats Directive, *C. jankae* is a species of high conservation importance at both regional and European levels (Anastasiu and Negrean 2008; European Environment Agency, 2007-2012; Bilz, 2011).

This study aimed to: (1) update the chorology of *C. jankae* and characterise the habitat ecology of its currently confirmed populations; (2) characterise variation in environmental conditions, vegetation composition, population structure, reproductive performance and morphological traits, together with the main anthropogenic pressures affecting the populations; and (3) model the current potential distribution and habitat suitability of the species using MaxEnt and evaluate the local conservation condition of the confirmed populations.

By integrating intraspecific morphological variation and reproductive performance with environmental conditions and community composition, this study provides a population-level functional ecological perspective on the persistence of a narrow endemic plant in fragmented steppe habitats.

The study tested the following hypotheses:

Based on the reported restricted and fragmented distribution of *C. jankae* (Bilz, 2011) and its association with calcareous, xerothermic habitats (Petrova, 2007), we hypothesised that (H1) currently confirmed populations are geographically isolated and primarily associated with fragmented, open calcareous steppe habitats.

Given the anthropogenic pressures reported for the species (Bilz, 2011) and the vulnerability of steppe communities to land-use change and woody vegetation encroachment (Shumilovskikh et al., 2021), we hypothesised that (H2) differences in population structure and reproductive performance are associated with variation in local environmental conditions and with observed anthropogenic and habitat-related pressures.

As no previous species distribution model has been developed for *C. jankae*, we hypothesised that (H3) its current potential distribution is strongly associated with land-cover and substrate characteristics, with additional contributions from topographic and climatic factors, resulting in a spatially restricted distribution of highly suitable habitat within the study area.

## 2 Material and methods

### 2.1 Study area

The study on *C. jankae* was conducted in Dobrogea, southeastern Romania and northeastern Bulgaria (**Figure 1**), during the 2023–2025 field seasons. The study region includes the Romanian counties of Constanţa and Tulcea and the Bulgarian administrative districts of Silistra, Dobrich, Varna, Shumen, and Razgrad. The investigated locations in Romania are characterised by a slightly warmer climate than those in Bulgaria, where June precipitation is higher and mean June temperature is lower (Fick and Hijmans, 2017). Elevations are higher at the Bulgarian sites (ranging from 61 to 360 m a.s.l.), than at the Romanian sites (ranging from 21 to 109 m a.s.l.). The main soil types recorded at the investigated sites were Calcic Chernozems and Calcic Kastanozems (IUSS Working Group WRB, 2022). The total study area covered 31,924.25 km^2^.

**Figure 1:**
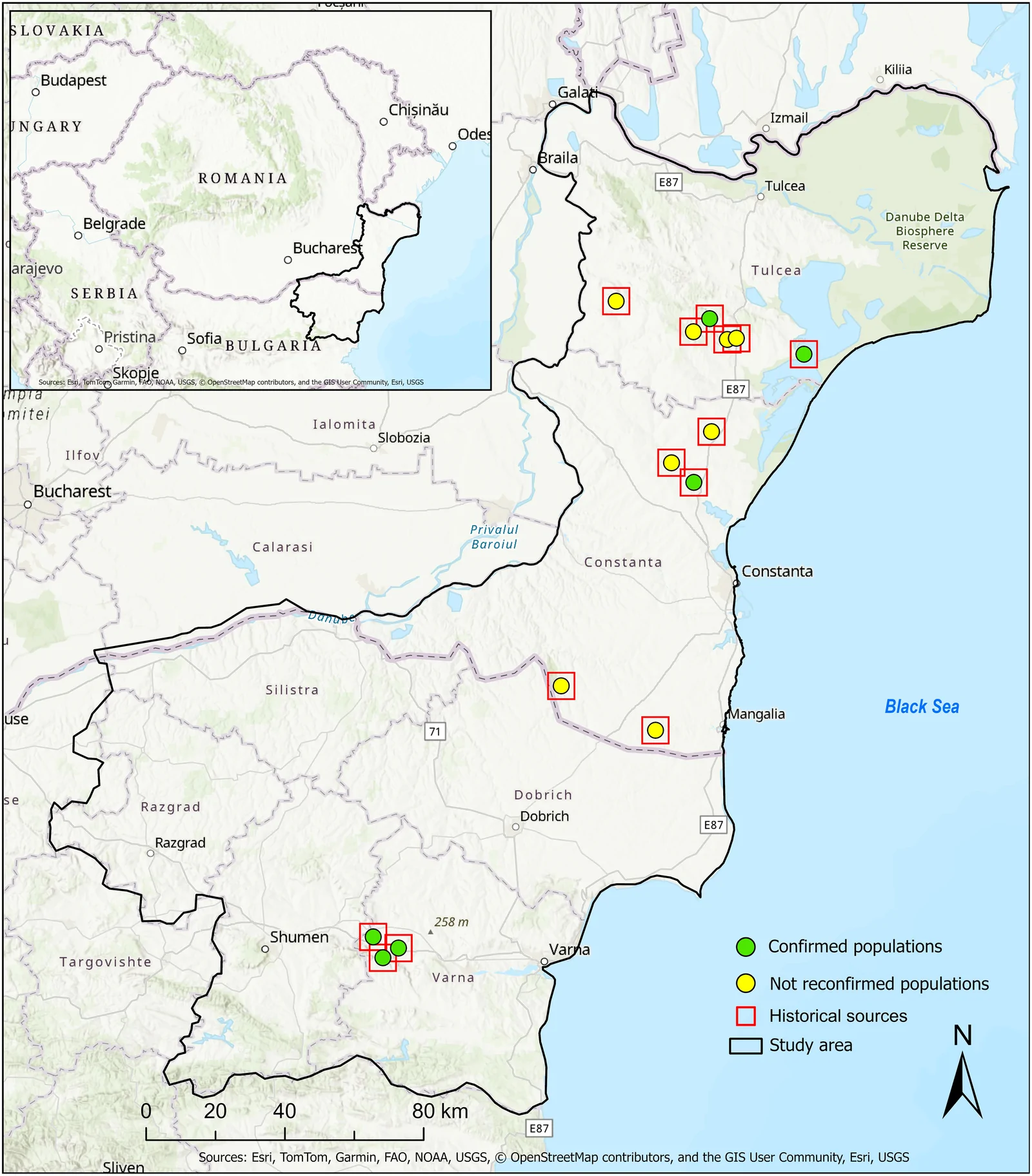
Historical and currently confirmed populations of *Centaurea jankae* in the study area. Green circles indicate populations confirmed during the 2023–2025 surveys, yellow circles indicate historical populations not reconfirmed, and red squares indicate historical sources. Coordinate reference system: [WGS 1984, EPSG:4326] (Map design: Nicu Alexandru Gîlea).

### 2.2 Study species

*Centaurea jankae* (**Figure 2**) is a perennial herb with a long, thickened rhizome. The young vegetative organs are arachnoid-pubescent, becoming glabrescent with age. The stems are solitary or several, 30–120 cm tall. They are robust, edged, striated (furrowed), simple or branched. The basal and lower cauline leaves are long-petiolate, 7–15 cm long, and have an oblong-ovate, bipinnatisect blade (Prodan and Nyárády, 1964; Dihoru and Negrean, 2009). The middle cauline leaves are pinnatisect, whereas the upper cauline leaves are reduced and may bear sessile, often abortive capitula in their axils. The leaf segments are patent, acute, entire, narrowly linear and cartilaginously mucronate; they broaden towards the apex and are usually approximately 1.5 mm wide, occasionally reaching 4 mm (Petrova, 2007).

**Figure 2:**
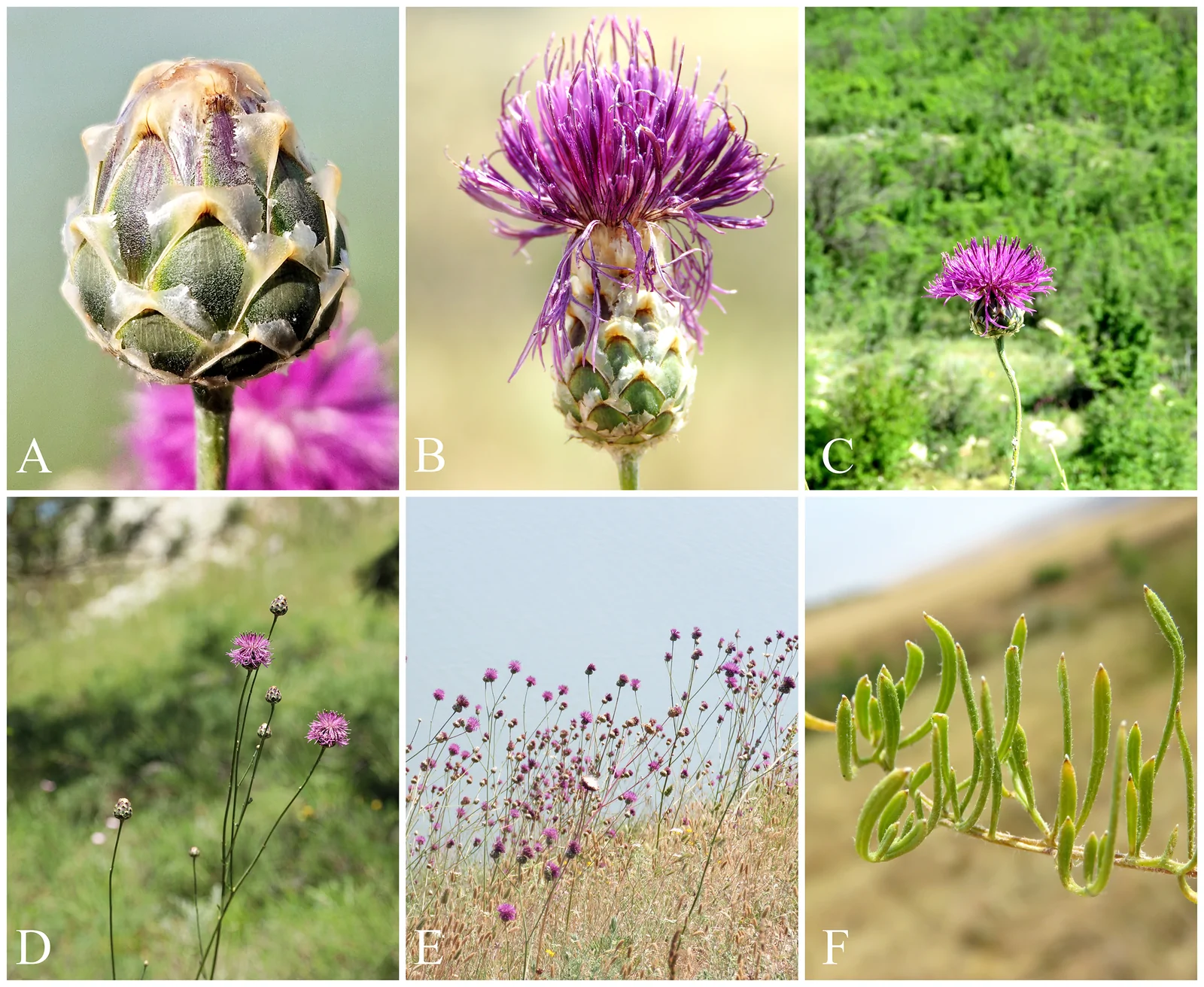
*Centaurea jankae*: **(a–c)** – capitula; **(d, e)** – habitus; **(f)** – leaf (Photographs: Simona Dumitriţa Chirilă).

The inflorescence consists of a solitary capitulum (calathidium) borne on a 5–10 cm-long peduncle; the capitulum is ovoid to subglobose (Prodan and Nyárády, 1964; Petrova, 2007). The achenes are pubescent and 5–5.5 mm long, while the pappus is either shorter than or approximately equal in length to the achene. The flowers are purple, and the marginal florets are radiating and glabrous. The involucral bracts are green and multistriate; the outer bracts are triangular-ovate, the middle bracts are ovate, and the inner bracts are ovate-lanceolate (Prodan and Nyárády, 1964).

The involucral appendages are scarious-membranous and erose. The appendages of the outer bracts are rounded or ovate, whereas those of the middle bracts are semilunar and have a membranous portion 2–3 mm wide. The appendages bear a small central brown macule (Prodan and Nyárády, 1964; Petrova, 2007).

*Centaurea jankae* generally flowers from May to June, while fruiting occurs from June to July (Petrova, 2007).

### 2.3 Environmental variables

To characterise the environmental conditions associated with *C. jankae* populations, variables describing topography, climate and soil properties were analysed.

#### 2.3.1 Topographic and climatic variables

The topographic variables included aspect, slope (°) and elevation (m a.s.l.). These variables were recorded in the field using OsmAnd version 4.7.17, Stare GPS version 11.4.316 and Clinometer version 3.0 applications.

The climatic variables included mean June temperature (°C), June precipitation (mm), annual mean temperature (BIO1, °C), temperature seasonality (BIO4, %), maximum temperature of the Warmest Month (BIO5, °C), minimum temperature of coldest month (BIO6, °C), temperature annual range (BIO7, °C), mean temperature of warmest quarter (BIO10, °C), annual precipitation (BIO12, mm), precipitation of driest month (BIO14, mm), precipitation seasonality (BIO15, CV%), precipitation of driest quarter (BIO17, mm) and precipitation of the warmest quarter (BIO18, mm). These variables were obtained from the WorldClim database (Fick and Hijmans, 2017). The June-specific variables were used exclusively to characterise the climatic conditions associated with the surveyed populations and were not included as predictors in the MaxEnt model version 3.4.4 (Phillips et al., 2025). June was selected because it corresponds to an ecologically relevant period for the flowering and early reproductive development of *C*. *jankae*. The bioclimatic variables were used to represent broader annual and seasonal climatic patterns.

Climatic aridity was additionally characterised using the monthly De Martonne aridity indices ( $I_{DMa}$ and $I_{DMm}$, respectively). The indices were calculated according to Quan et al. (2013). The annual index was calculated as follows:


$$I_{DMa}=\frac{P_{a}}{\left(T_{a}+10\right)}$$

where $P_{a}$ is annual precipitation (mm) and $T_{a}$ is mean annual temperature (°C). The monthly index was calculated using monthly precipitation $P_{m}$ and mean monthly temperature $T_{m}$.


$$I_{DMm}=\frac{12P_{m}}{\left(T_{m}+10\right)}$$

#### 2.3.2 Soil sampling and edaphic variables

A total of 38 soil samples were collected and analysed from a subset of the 52 phytocoenological plots representing the five populations included in the vegetation dataset: Jurilovca–Capul Doloşman, Palazu Mic, Slava Rusă, Nevsha and Ravna. Each vegetation plot covered 100 m^2^, and one soil sample was collected from a depth of 0–15 cm within each selected plot. The edaphic variables analysed were organic carbon content (OrgC %), calcium content (Ca, mg/100g soil), soil pH, total phosphorus (mg/100g soil), ammonium nitrogen (N-NH_4_), nitrite nitrogen (N-NO_2_), nitrate nitrogen (N-NO_3_), and total and organic nitrogen contents (mg/100g soil). To determine the soil variables, molecular absorption spectrometry, volumetric, potentiometric, and inductively coupled plasma–mass spectrometry (ICP-MS) methods were used. All mass-based concentrations are reported relative to 100 g of dry soil. Soil parameters were determined according to STAS 7184/21-82, SR ISO 10390:1999, STAS 7184/14-79, SR ISO 14255:2000, and SR ISO 11261:2000 (Romanian Standards Association, 1979, 1982, 1999, 2000a, 2000b), while calcium was determined using U.S. EPA Method 6020A (U.S. Environmental Protection Agency, 2007). Soil types were classified according to the World Reference Base for Soil Resources (IUSS Working Group WRB, 2022).

#### 2.3.3 Vegetation sampling and habitat classification

To identify the plant associations in which *C. jankae* occurs, 52 phytocoenological relevés were conducted, each covering an area of 100 m^2^. The Braun–Blanquet cover-abundance scale (1964) adapted according to Cristea et al. (2004), was used as follows: r (0.05%), + (0.5%), 1 (5%), 2 (17.5%), 3 (37.5%), 4 (62.5%), and 5 (87.5%). The nomenclature of plant associations followed Chifu et al. (2014), while the nomenclature of higher syntaxa followed Mucina et al. (2016). EUNIS habitat types were identified using the expert-based classification system developed by Chytrý et al. (2020). The vegetation and soil datasets comprised five of the six confirmed populations (Jurilovca–Capul Doloşman, Palazu Mic, Slava Rusă, Nevsha, and Ravna); the Venchan population was characterised demographically only and was therefore not included in the relevé-based vegetation and environmental analyses.

Differences in floristic composition between the five investigated populations were assessed using a site-species matrix obtained from the 52 phytocoenological relevés. Braun-Blanquet cover abundance values were converted to the corresponding mean percentages and square root transformed. NMDS was performed on this square-root-transformed community matrix using Bray–Curtis dissimilarities with the metaMDS function in the vegan package (k = 2, try = 50, trymax = 999, autotransform = FALSE; random seed = 123). Population-level 95% confidence ellipses were generated from site scores using ordiellipse (kind = “se”, conf = 0.95).

Differences in floristic composition between populations were tested using multivariate permutational analysis of variance (PERMANOVA) with 9,999 permutations. Homogeneity of multivariate dispersion was assessed using the betadisper procedure and a permutation test. Following a significant overall PERMANOVA, pairwise comparisons between populations were performed, and the resulting p-values ​​were adjusted using the Holm method. The complete NMDS/PERMANOVA workflow was repeated after excluding *C. jankae* as a sensitivity analysis.

### 2.4 Chorological assessment

The chorology of *C. jankae* was assessed using data compiled from published scientific literature, floristic monographs, herbarium records and field observations. Historical occurrences and their supporting published sources or herbarium vouchers are documented in **Supplementary Tables 1**, **4**. Historical records reported in the literature and herbarium collections were verified during field surveys conducted between 2023 and 2025. Field investigations were carried out at previously reported locations and in additional areas containing potentially suitable habitat for the species. Each record was classified as historical, currently confirmed or not reconfirmed during the present surveys. All currently confirmed populations were georeferenced and used to produce updated distribution maps. The spatial distribution of the species was mapped using QGIS version 3.34.3 (QGIS Development Team, 2023), and the maps were subsequently edited in Inkscape version 1.3.2 (https://inkscape.org/; Inkscape Project, 2023).

### 2.5 Morphological traits and population structure

In each *C. jankae* population, morphological and demographic parameters were recorded during the 2025 field surveys. Morphological parameters included plant height, leaf length and width, and the number of leaves per plant. Plant height was measured from the base of the stem to the uppermost capitulum. Leaf length and width were measured on a median leaf using a metal measuring tape, while the number of leaves was determined by direct counting. In addition to these morphological measurements, the number of capitula in anthesis and the number of buds per plant were recorded as individual-level reproductive traits where available.

Total population size was determined through a complete census of each delimited population. Local demographic data were additionally recorded in 100 m^2^ plots. Within each plot, the total number of individuals and the numbers of flowering and vegetative individuals were recorded by direct counting. Population density was expressed as the number of individuals per 100 m^2^. When more than one plot was surveyed, the density range was calculated using the minimum and maximum plot-level values. The proportion of flowering individuals was calculated as follows:


$$F=\frac{N_{f}}{N_{t}}\times 100$$

where $N_{f}$ is the number of flowering individuals, and $N_{t}$ is the total number of individuals recorded in the corresponding population or in the sampled plots.

Demographic assessments were conducted in May and June 2025, during the flowering period of *C. jankae*. Individual flowers or capitula were not counted as part of the plot-level demographic census. Instead, an individual was classified as flowering when it bore at least one open capitulum. Within each 100 m^2^ plot, the total number of individuals and the numbers of flowering and vegetative individuals were recorded simultaneously.

### 2.6 Temporal trend assessment

Previous population estimates were compiled from published studies and management plans. These estimates were considered directly comparable with the 2025 data only when they referred to the same population.

When two directly comparable estimates were available, the mean annual rate of population change was calculated as follows:


$$r=[{\left(\frac{N_{t}}{N_{0}}\right)}^{\frac{1}{t}}1]\times 100$$

where $N_{0}$ is the population estimate at the beginning of the comparison period, $N_{t\;}$ is the estimate at the end of the period, and t is the number of years between assessments.

Population trends of *C. jankae* were classified as increasing, stable, decreasing or unknown. Increasing, stable or decreasing trends were assigned only when repeated estimates were obtained using demonstrably comparable survey areas, counting units and census methods. When the survey extent, counting units or census methods differed or were insufficiently studied, the annual rate of change was not calculated (NC), and the population trend was classified as unknown (XX).

### 2.7 Local conservation-condition assessment

As the formal conservation condition assessment under Article 17 of the Habitats Directive is conducted at the biogeographical-region level of each Member State, the assessments presented in this study were treated as local population-level assessments. They were informed by the Article 17 framework but not equivalent to the official assessment (Council of the European Communities, 1992; European Commission, 2017).

Four components were assessed: (i) population condition, based on abundance, density and reproductive structure; (ii) local occupancy and spatial fragmentation; (iii) habitat condition, considering the continuity of open steppe habitat, grazing, damage to inflorescences, vegetation succession, woody vegetation and pine encroachment, shading and other observed disturbances; and (iv) future prospects, based on identified pressures and existing conservation measures.

Each component was classified as favourable (FV), unfavourable–inadequate (U1), unfavourable–bad (U2), or unknown (XX). FV was assigned when population and habitat conditions were adequate and no major ongoing pressures were identified; U1 was assigned when moderate limitations or persistent pressures were recorded; U2 was assigned in case of severe population or habitat deterioration. XX was used when the available information was insufficient.

Because no validated numerical thresholds are available for *C. jankae*, abundance, density and flowering proportion were interpreted alongside information on occupancy, habitat condition and local pressures. The overall classification was assigned conservatively and was determined by the most unfavourable component. Confidence was classified as high, medium or low according to the completeness, comparability and quantitative basis of the available data.

### 2.8 Spatial modelling

#### 2.8.1 Occurrence data and spatial thinning

For spatial modelling, an initial dataset of 293 georeferenced presence records was compiled from the occurrence data documented during the chorological assessment (Section 2.4). These records included data from the 2023–2025 field surveys and historically studied locations.

To reduce spatial sampling bias resulting from the uneven distribution of occurrence records, spatial thinning was performed in R version 4.5.0 using the spThin package. Spatial thinning is commonly applied in species distribution modelling to reduce spatial autocorrelation among occurrence records and to minimize the over-representation of environmental conditions in intensively sampled areas (Aiello‐Lammens et al., 2015).

A minimum distance threshold of approximately 100 m was applied between occurrence records to match the spatial resolution of the environmental predictor rasters. The thinning algorithm implemented in spThin iteratively removed records located within the specified minimum distance while retaining a subset of occurrences that satisfied the distance criterion. After spatial thinning, 27 occurrence records (**Supplementary Table 2**, **A1**) were retained and used to model the distribution of *C. jankae* in Dobrogea (**Figure 3**). Although the number of occurrence records was relatively small, previous studies have shown that MaxEnt provides reliable predictions even with limited sample sizes, particularly when occurrence records are carefully filtered and model complexity is appropriately controlled (Hernandez et al., 2006; Pearson et al., 2007).

**Figure 3:**
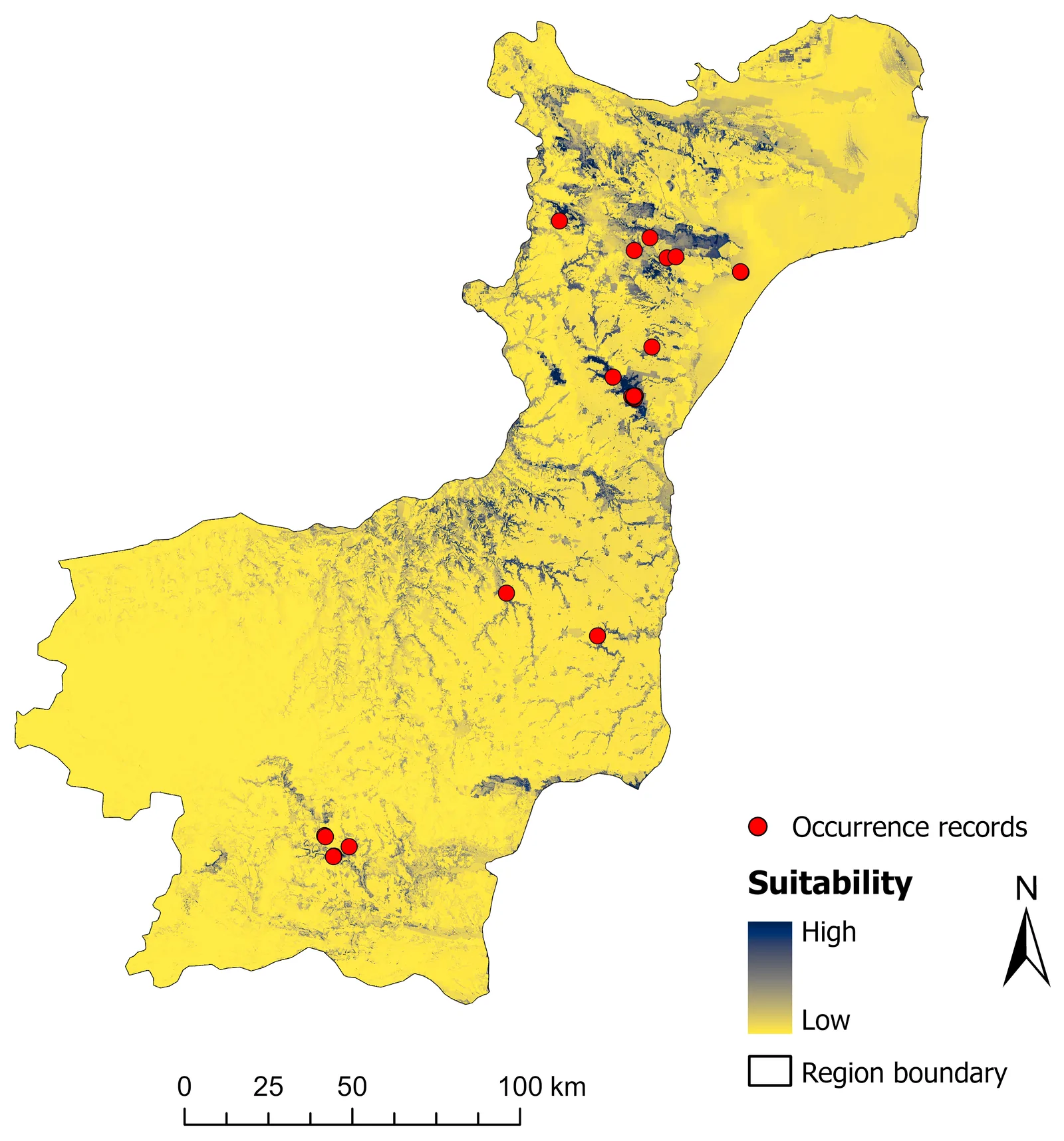
Current potential distribution of *Centaurea jankae* predicted by MaxEnt. Occurrence records used for model calibration are overlaid on the predicted habitat suitability map.

#### 2.8.2 Species distribution modelling: environmental data and preprocessing

Species distribution modelling was based on 27 occurrence records retained after the spatial thinning procedure described above and on a set of environmental predictors representing climate, topography, land cover and soil conditions. The 19 bioclimatic variables were obtained from the WorldClim version 2.1 database at a native spatial resolution of 30 arc-seconds, corresponding to approximately 1 km at the Equator (Fick and Hijmans, 2017) (**Table 1**). Topographic predictors, including elevation, slope, and aspect, were acquired directly from the Digital Elevation Model over Europe (EU-DEM) dataset provided by the European Environment Agency at a spatial resolution of 30 m. The slope layer is stored in the SLOP format, where pixel values are encoded as Digital Numbers (DN) ranging from 0 to 250 rather than as angular degrees; these DN values can be converted to slope angles using the standard conversion function [Slope (°) = arccos(DN/250) × 180/π] (European Environment Agency, 2016). Land use/land cover (LULC) data were obtained from the ESA WorldCover 2021 v200 dataset, developed by the European Space Agency (ESA), at a spatial resolution of 10 m (Zanaga et al., 2022). Soil major groups were obtained from the European Soil Database v2 Raster Library (FAO90LV1), available through the European Soil Data Centre (ESDAC) at a native spatial resolution of approximately 1 km (Panagos et al., 2022). Soil pH and soil organic carbon (SOC) were obtained from the SoilGrids database at a spatial resolution of 250 m (Poggio et al., 2021).

**Table 1:** Environmental variables used for spatial modelling of *Centaurea jankae*.

Variable abbreviation	Description	Unit	Resolution
BIO1	Annual Mean Temperature	°C	30 arc-seconds (~1 km at the Equator)
BIO2	Mean Diurnal Range (Mean of monthly (max temp - min temp))	°C	
BIO3	Isothermality (BIO2/BIO7) (×100)	–	
BIO4	Temperature Seasonality (standard deviation ×100)	°C	
BIO5	Max Temperature of Warmest Month	°C	
BIO6	Min Temperature of Coldest Month	°C	
BIO7	Temperature Annual Range (BIO5 - BIO6)	°C	
BIO8	Mean Temperature of Wettest Quarter	°C	
BIO9	Mean Temperature of Driest Quarter	°C	
BIO10	Mean Temperature of Warmest Quarter	°C	
BIO11	Mean Temperature of Coldest Quarter	°C	
BIO12	Annual Precipitation	mm	
BIO13	Precipitation of Wettest Month	mm	
BIO14	Precipitation of Driest Month	mm	
BIO15	Precipitation Seasonality (Coefficient of Variation)	%	
BIO16	Precipitation of Wettest Quarter	mm	
BIO17	Precipitation of Driest Quarter	mm	
BIO18	Precipitation of Warmest Quarter	mm	
BIO19	Precipitation of Coldest Quarter	mm	
Elevation	Elevation	m a.s.l.	
Slope	Slope	DN (0–250)	30 m
Aspect	Aspect	°	30 m
LULC	Land use/cover	categorical	10 m
Soil	Soil major group code of the STU from the 1974 (modified CEC 1985) FAO-UNESCO Soil Legend.	categorical	approximately 1 km native resolution
pH	soil pH (0–5 cm)	pH*10	250 m
SOC	Soil organic carbon (0–5 cm)	dg/kg	250 m

All environmental rasters were clipped to the Dobrogea study-area boundary, reprojected to a common geographic coordinate reference system (WGS 84; EPSG:4326), aligned to the same spatial extent, and resampled to a common grid with a cell size of 0.00107133° (approximately 100 m in the study area). The use of a common grid ensured spatial correspondence among the predictor layers and the occurrence-data grid. Resampling harmonised the raster geometry but did not increase the native spatial information content of the WorldClim and soil datasets. The resulting rasters were exported in ASCII format for use in MaxEnt. All spatial-data processing was performed in ArcGIS Pro version 3.6.0.

The potential distribution of *C*. *jankae* was modelled using the MaxEnt presence-background modelling algorithm (Phillips et al., 2006; Phillips and Dudík, 2008).

#### 2.8.3 Selection of environmental variables

Variable selection aimed to reduce multicollinearity, limit model complexity and retain ecologically interpretable predictors. An initial MaxEnt model was fitted using all 26 candidate environmental variables (**Supplementary Table 2**; **Supplementary Figures A1–A4**; **Supplementary Table A2**). Pairwise correlations among the continuous predictors were subsequently assessed in R using Pearson’s correlation coefficient (**Supplementary Tables 2**, **A4**; **Supplementary Figure A5**). Pairs of continuous variables with ∣r∣>0.7 were considered highly correlated and were not retained together in the final predictor set (Dormann et al., 2013).

When two continuous variables were highly correlated, the variable with greater ecological relevance and a higher contribution to the initial MaxEnt model was retained (Duan et al., 2025).

Pearson correlation analysis was applied exclusively to continuous environmental predictors. Land use/Land cover (LULC) and soil type were excluded from the Pearson correlation analysis because they were treated as nominal categorical predictors in MaxEnt. No Pearson correlation coefficients were therefore calculated between these categorical predictors and the continuous environmental variables.

As an additional diagnostic, multicollinearity among the retained continuous predictors was evaluated using the Variance Inflation Factor (VIF) (**Table 2**), calculated in R (version 4.5.0) with the usdm package (Naimi et al., 2014). VIF analysis was applied to BIO7, BIO18 and slope, whereas the categorical predictors LULC and soil type were excluded from this analysis. A threshold of VIF < 5 was used to indicate acceptable levels of multicollinearity. The predictor set retained for the model comprised LULC, soil type, BIO18, BIO7 and slope.

**Table 2:** Pearson correlation coefficients and variance inflation factors (VIF) for the continuous predictors retained in the final SDM.

Predictor	BIO7	BIO18	Slope	VIF
BIO7	1	0.090	0.476	1.309
BIO18		1	0.380	1.183
Slope			1	1.517

#### 2.8.4 Model calibration and evaluation

The MaxEnt model was calibrated through a two-step procedure aimed at selecting the most appropriate model settings while minimizing overfitting. Model performance was assessed using the mean test Area Under the Receiver Operating Characteristic Curve (test AUC), the standard deviation of test AUC (SD), the 10-percentile training presence test omission rate, the test gain, and the difference between training and test AUC across replicate runs.

In the first step, the optimal number of cross-validation replicates was determined. Using the final set of five environmental predictors and the 27 occurrence records, the model was repeatedly calibrated with replicate numbers ranging from 2 to 10. For all candidate models, only Linear (L) and Quadratic (Q) feature classes were enabled, as these simpler feature types are recommended for small sample sizes because they reduce model complexity and the risk of overfitting (Merow et al., 2013; Morales et al., 2017). All remaining MaxEnt settings were kept at their default values. Among the tested configurations, five cross-validation replicates provided the best compromise between predictive performance and model stability, yielding the highest mean test AUC (0.928) while maintaining an omission rate of 0.14. Therefore, five-fold cross-validation was adopted for all subsequent analyses (**Supplementary Table 2**, **A5**).

In the second step, the regularization multiplier (RM) was optimized by testing values ranging from 0.25 to 2.00 (0.25, 0.50, 0.75, 1.00, 1.25, 1.50, 1.75, and 2.00). Model performance varied only slightly among the tested RM values (test AUC = 0.922–0.930). The optimal model was obtained with RM = 0.75, which achieved the highest mean test AUC (0.930), a relatively low standard deviation (0.056), a test gain of 2.208, and a small difference between training and test AUC, indicating a good balance between predictive accuracy and model generalization (**Supplementary Table 2**, **A6**). Because mean test AUC differed only marginally across the tested regularization multipliers (0.922–0.930; **Supplementary Table 2**, **A6**), the final value was not selected on the basis of AUC alone. Instead, RM = 0.75 was retained because it provided the highest mean test AUC while maintaining a relatively small training–test AUC difference and a high test gain.

The optimal calibration settings selected for the final MaxEnt model (version 3.4.4) therefore consisted of Linear and Quadratic feature classes, a regularization multiplier of 0.75, five cross-validation replicates, and a maximum of 10,000 background points. Land-use/land-cover (LULC) and soil type were specified as categorical predictors. The output format was set to cloglog, raster predictions were exported as ASCII files, and all remaining MaxEnt parameters were left at their default values.

#### 2.8.5 Modelling the current distribution

Using the optimal settings identified during the calibration procedure, the final MaxEnt model was built to estimate the current potential distribution of *C. jankae*. The model incorporated the 27 presence records (**Supplementary Table 2**, **A1**) and the five environmental predictors (LULC, Soil type, Slope, Bio18, Bio7) retained after the variable-selection procedure. Model calibration was performed across the complete study area using environmental background points rather than confirmed absence records. Model performance was evaluated based on the mean test AUC, its standard deviation, and the 10-percentile training presence test omission rate across the five cross-validation replicates. The resulting prediction represents the current potential distribution of *C. jankae* under contemporary environmental conditions.

#### 2.8.6 Habitat suitability assessment for *C. jankae*

Based on the SDM, we determined habitat suitability (HS), used to interpret and classify the degree of habitat favourability within the Dobrogea. For this, we loaded the resulting file with the suffix “average” into ArcGIS Pro v3.6.0, which contains the spatial distribution of species occurrence probability in cloglog format (values between 0 and 1). The decision threshold used to separate suitable from unsuitable habitats was the maximum sensitivity–specificity cloglog threshold (0.20), derived from ROC analysis and obtained across the 5 cross-validation replicates (Qi et al., 2025). We then applied a raster reclassification process using the *Reclassify* tool to transform the continuous raster into a categorical raster, reflecting distinct levels of ecological suitability. Areas were classified into four categories (Acharya et al., 2023; Duan et al., 2025) as follows: < 0.2—unsuitable, 0.2–0.4—low suitability, 0.4–0.6—medium suitability, ≥ 0.6—high suitability.

### 2.9 Statistical analysis

Statistical analyses and data visualisation were performed in R version 4.5.0 (R Core Team, 2025). Differences among populations in soil properties and nitrogen forms were tested separately for each variable using Kruskal–Wallis tests. When the overall Kruskal–Wallis test was significant, pairwise differences between populations were assessed using Dunn’s *post-hoc* test with Bonferroni correction for multiple comparisons. Statistical significance was set at p < 0.05. Floristic composition was analysed using the vegan package version 2.7-2, as described above. The graphs (polar plots, violin plots and boxplots) were generated using the ggplot2 package (Wickham, 2016). Final editing and layout adjustments were performed in Inkscape version 1.3.2 (https://inkscape.org/).

Because the vegetation and soil datasets represented only three populations in Romania and two in Bulgaria, no inferential country-level comparisons were performed. Differences between Romanian and Bulgarian populations are therefore described descriptively only.

## 3 Results

### 3.1 Environmental conditions

The five populations of *C. jankae* are distributed along a climatic gradient, from the hottest and driest conditions in Romania to the coolest and wetter ones in Bulgaria. The Jurilovca population occupies the arid end of the gradient, being characterised by the highest temperature in June, the lowest precipitation amounts, and the lowest value of the De Martonne aridity index. At the opposite extreme, the Nevsha population recorded the highest precipitation amounts, both annually and in June, lower temperatures, and the highest value of the De Martonne index. The Romanian populations (Jurilovca, Palazu Mic, and Slava Rusă) were grouped in the hot and arid zone, and the Bulgarian ones (Nevsha and Ravna) in the wetter and cooler one. The transition between the two groups is gradual, not abrupt: the Slava Rusă population presented wetter conditions than the Jurilovca and Palazu Mic populations (**Figure 4**).

**Figure 4:**
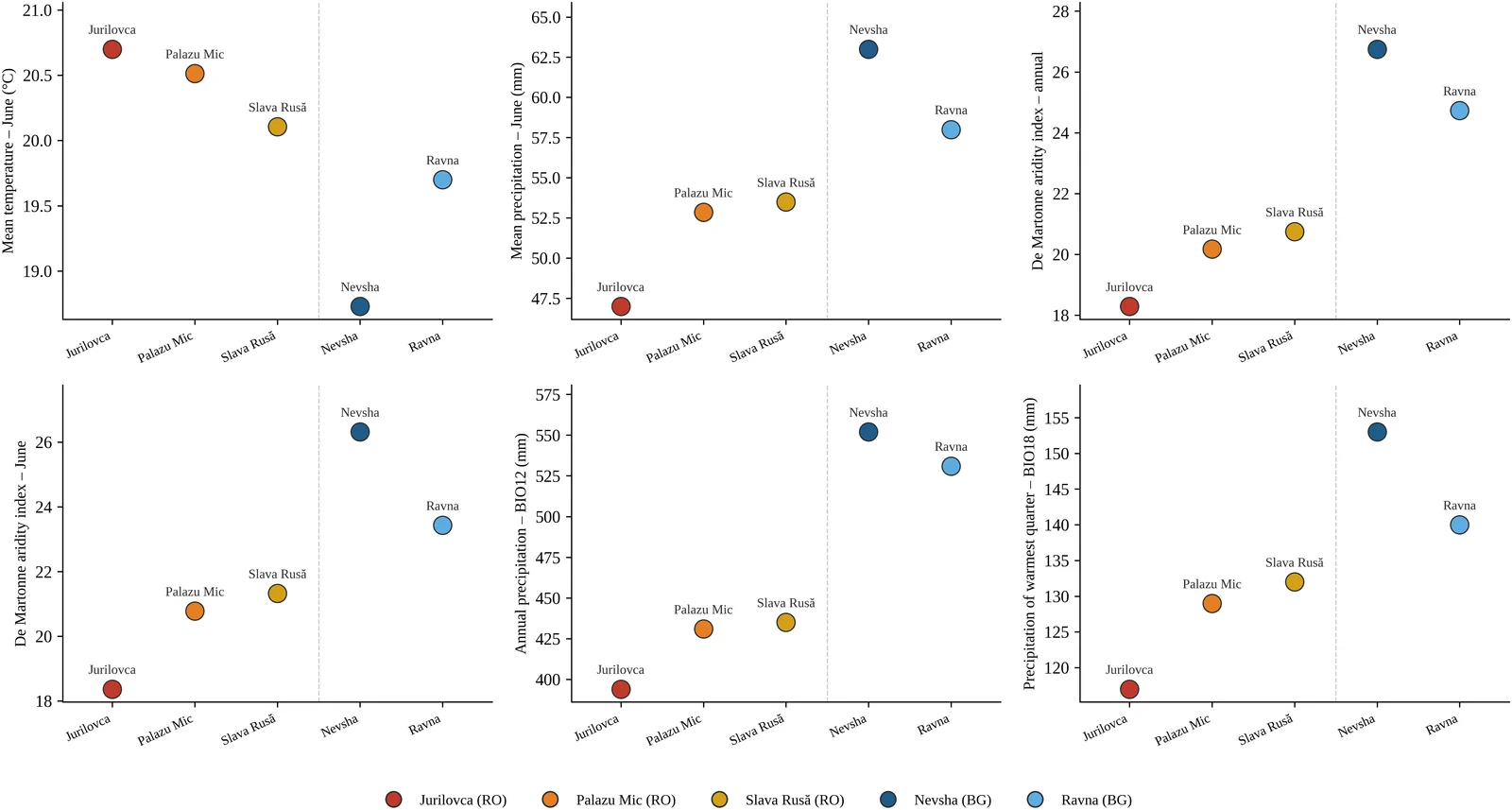
Bioclimatic variation among *Centaurea jankae* populations (n = 5). Each point represents one population; climatic values were extracted from WorldClim at the population level, as repeated plot-level values within the same raster cell would not constitute independent observations. Blue points represent Bulgarian populations and warm-coloured points represent Romanian populations. The annual and June De Martonne indices were calculated for each population using the formulas described in the Methods. The Venchan population was characterised demographically only and is therefore not shown.

*Centaurea jankae* was recorded on both gentle and steep slopes, including slopes exceeding 50°. Higher cover values tended to occur on moderately inclined slopes. Regarding aspect, *C*. *jankae* was recorded predominantly on sunny slopes, whereas lower cover values were generally observed on shaded slopes. Regarding elevation, *C. jankae* occurred mainly at low to moderate elevations, where comparatively high cover values were observed. However, at some Bulgarian sites, high cover values were also observed at elevations above 250 m a.s.l. (**Figure 5**).

**Figure 5:**
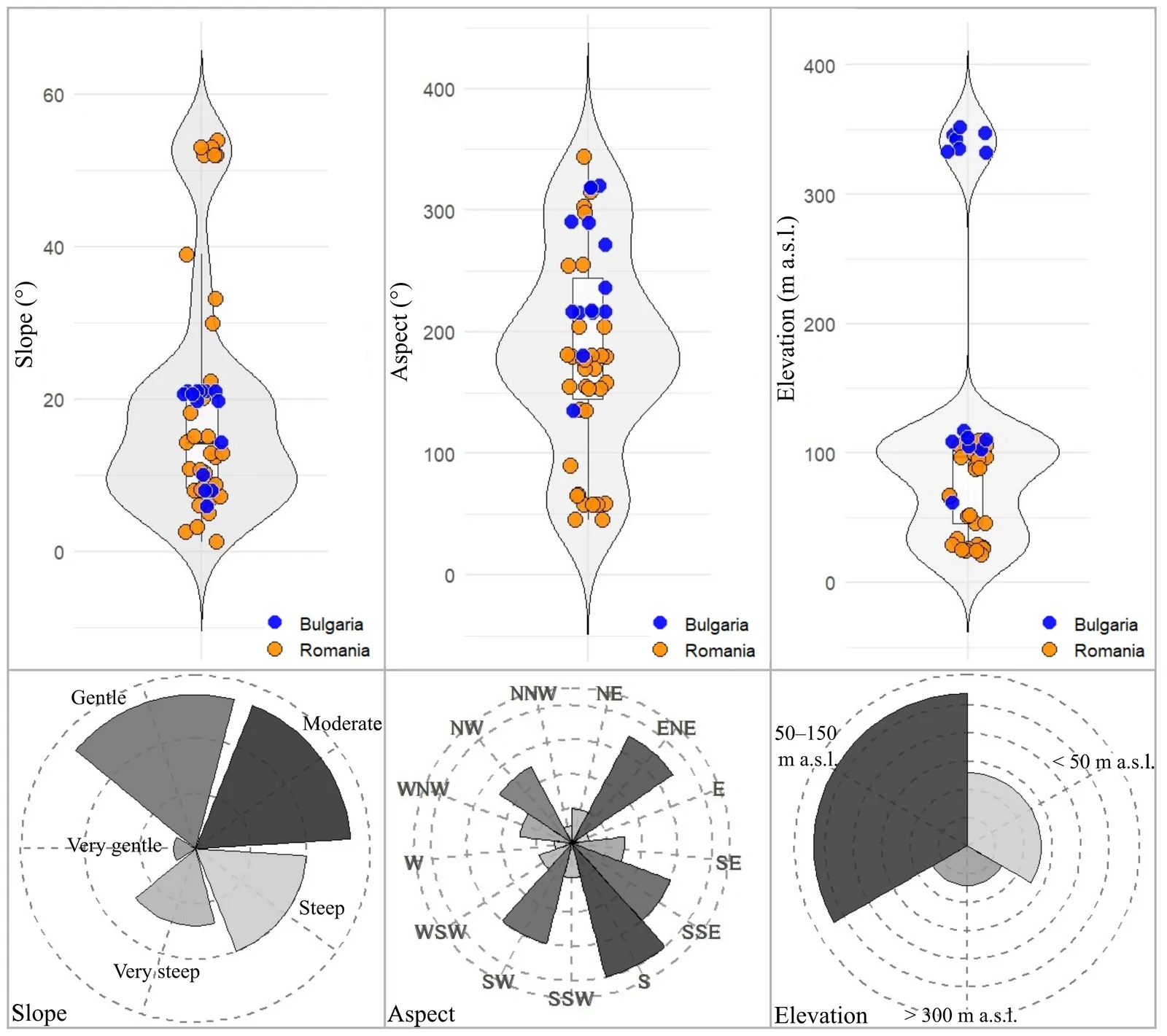
Variation of topographic factors associated with *Centaurea jankae.* Each point represents one sampled plot (n = 47; Romania, n = 33; Bulgaria, n = 14). Blue points represent Bulgaria and orange points represent Romania. Violin width represents the density of observations at a given value. The polar diagrams show the frequency of plots within predefined slope classes, aspect sectors and elevation classes; they do not represent the cover or frequency of *C. jankae*.

*Centaurea jankae* populations showed significant differences for most of the analysed soil properties, but the variation does not outline a clear separation between the Romanian and Bulgarian populations. In general, one or two populations showed different values, while the others remained relatively similar. Soil pH showed the largest differences between populations (p < 0.001). The soils from Slava Rusă were the least alkaline, followed by those from Palazu Mic, while the soils from Jurilovca, Nevsha and Ravna had higher pH values. In the case of phosphorus, the soils from Ravna differed in the lowest concentrations, with the other four populations having comparable values. The soils from Nevsha showed the lowest values of organic carbon and humus, and the soils from Slava Rusă were distinguished by a higher calcium content. For organic nitrogen, the overall Kruskal–Wallis test was significant, although no pairwise differences remained significant after Bonferroni correction. As such, Romanian populations tend to be associated with higher values of some soil fertility indicators, such as phosphorus, organic carbon and humus, while some Bulgarian populations present poorer and more alkaline soils (**Figure 6**).

**Figure 6:**
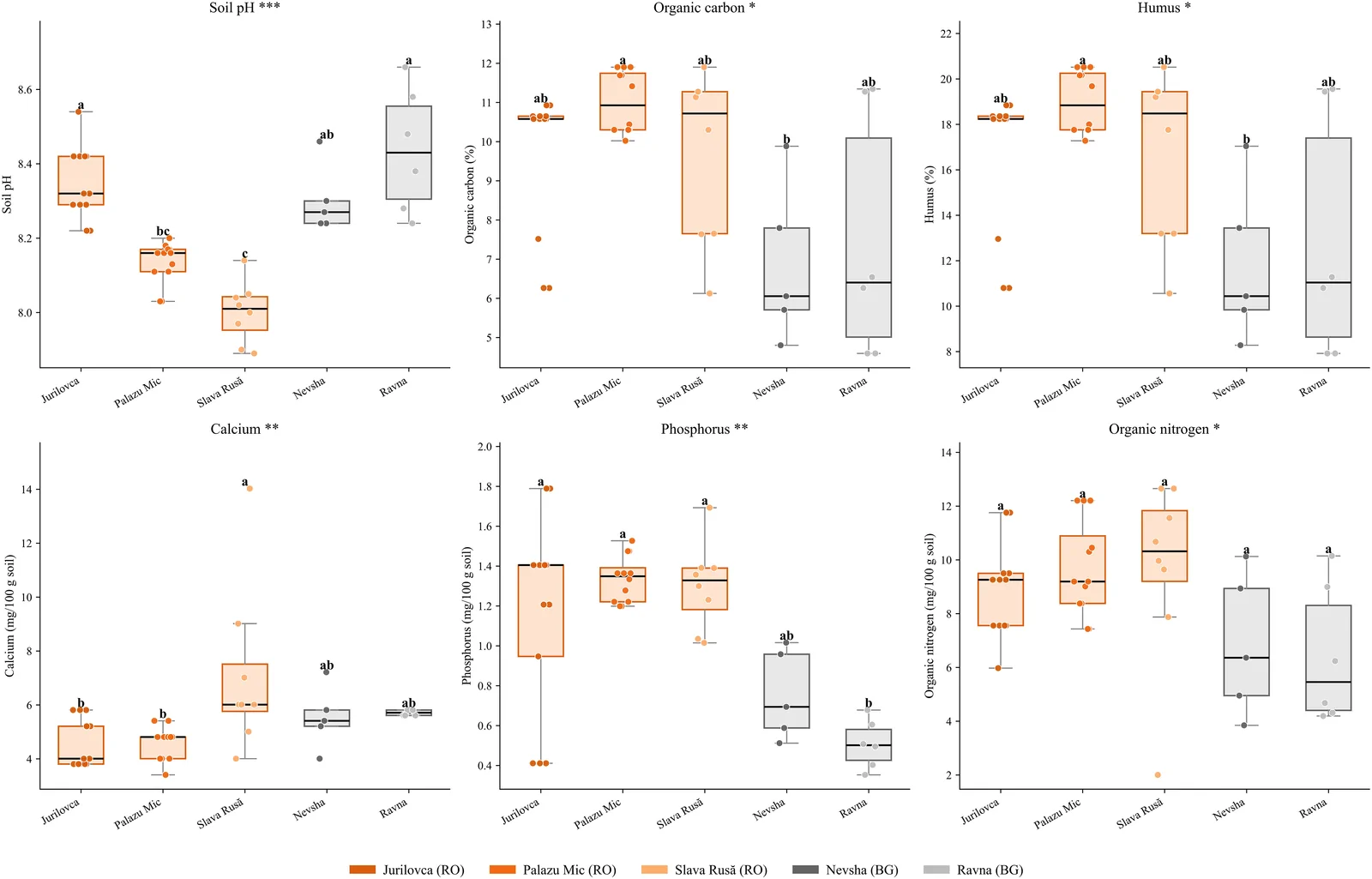
Soil properties among *Centaurea jankae* populations. Differences among populations were tested separately for each soil variable using Kruskal–Wallis tests. Where the overall test was significant, pairwise comparisons were performed using Dunn’s *post-hoc* test with Bonferroni correction. Populations sharing at least one letter did not show a significant pairwise difference (adjusted p > 0.05), whereas populations with no letters in common differed significantly (adjusted p < 0.05). Asterisks indicate the significance of the overall Kruskal–Wallis test: *p < 0.05, **p < 0.01, ***p < 0.001.

The indicators of mineral nitrogen forms showed significant differences between populations. In contrast, no significant differences were identified for total nitrogen between populations. Nitrate concentrations (NO_3_^−^ and N–NO_3_) were highest at Jurilovca and lowest at Palazu Mic. Ammonium concentrations reached their highest observed values at Slava Rusă. Nitrite concentrations were also comparatively high at Slava Rusă, whereas lower values were recorded at several other populations, particularly Nevsha. Overall, the mineral nitrogen forms showed significant among-population differences (**Figure 7**).

**Figure 7:**
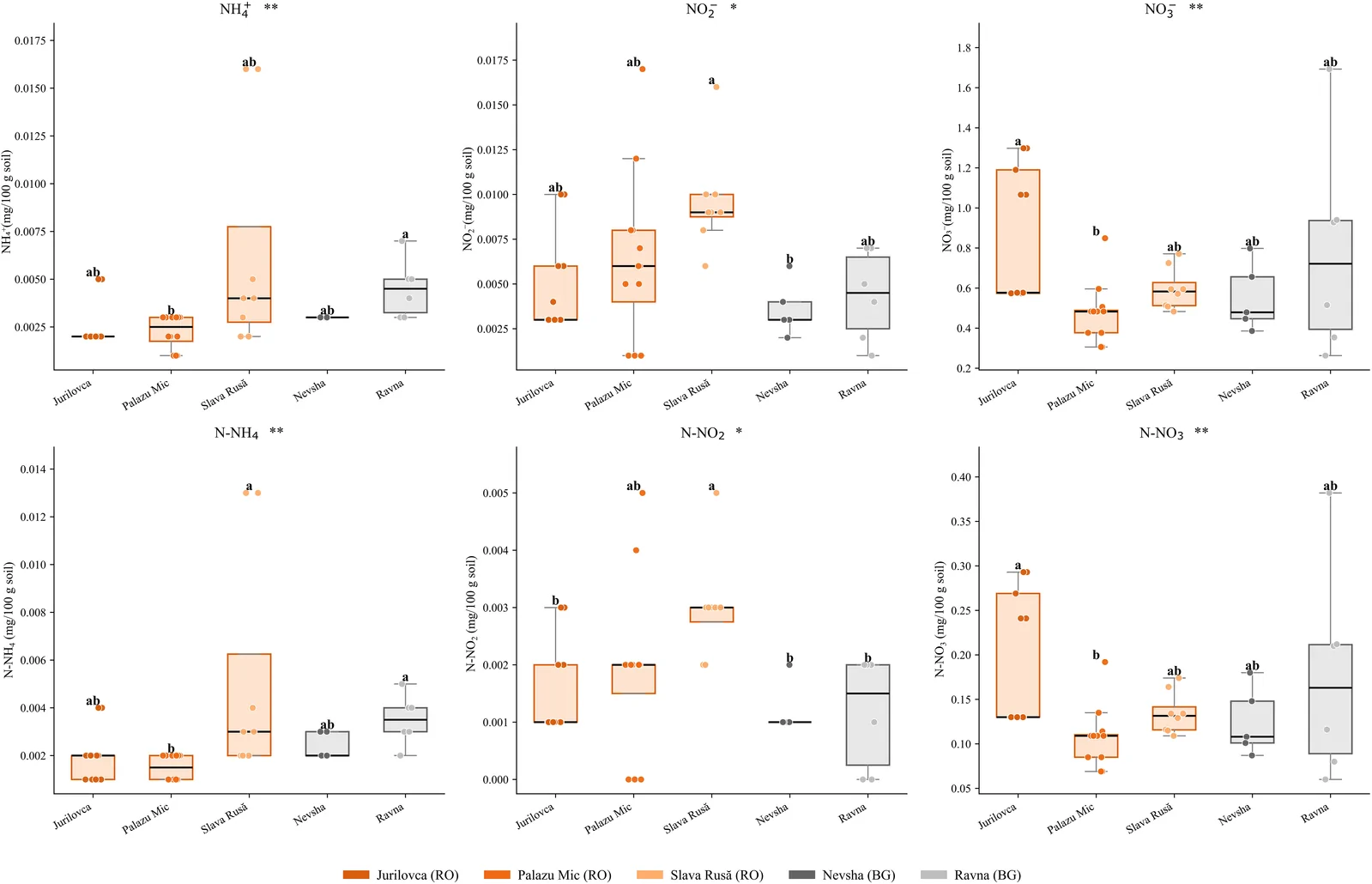
Soil nitrogen forms among *Centaurea jankae* populations. Differences among populations were tested separately for each nitrogen variable using Kruskal–Wallis tests. Where the overall test was significant, pairwise comparisons were performed using Dunn’s *post-hoc* test with Bonferroni correction. Populations sharing at least one letter did not show a significant pairwise difference (adjusted p > 0.05), whereas populations with no letters in common differed significantly (adjusted p < 0.05). Asterisks indicate the significance of the overall Kruskal–Wallis test: *p < 0.05, **p < 0.01, ***p < 0.001.

Populations of *C. jankae* in Romania were recorded under drier conditions than those in northeastern Bulgaria, as indicated by lower annual precipitation and De Martonne index values. Romanian populations were also associated with a greater annual temperature range and more pronounced temperature seasonality. In contrast, Bulgarian populations were characterised by a more uniform precipitation regime, including during the driest periods. Romanian populations occurred mainly at low elevations, whereas Bulgarian populations occurred at higher elevations (**Supplementary Table 3**).

In Romania, slopes were generally moderate and aspects varied, whereas southwestern aspects predominated at the Bulgarian sites. Soils in Romania were classified as Calcic Kastanozem and Calcic Chernozem and showed relatively higher contents of organic matter and nutrients (organic carbon, humus, phosphorus and organic nitrogen). In contrast, Bulgarian soils were mainly Calcic Chernozems, with lower organic matter contents, slightly higher pH values and of relatively higher mean nitrate concentrations.

### 3.2 Chorology

Field investigations revisited several historically reported locations in Romania and Bulgaria. The presence of *C. jankae* was confirmed at three locations in Romania (Palazu Mic, Jurilovca–Capul Doloşman, and Slava Rusă) and three in Bulgaria (Nevsha, Ravna, and Venchan). The species had previously been recorded in the grasslands of Cotu Văii and Cheile Dobrogei during field observations conducted in 2017, 2018 and 2020, but it was not reconfirmed at either locality during the 2023–2025 surveys. Other historically reported locations, including Dumbrăveni, Tariverde, Codru (Babadag Forest area) and Fântâna Oilor, were also not reconfirmed. The currently confirmed populations are geographically isolated and separated by extensive areas containing historical records at which the species was not detected during the present surveys (**Supplementary Table 4**).

#### 3.2.1 Description of populations

##### 3.2.1.1 Palazu Mic

The *C. jankae* population at Palazu Mic (Constanţa County, Romania) occurs at elevations of 45–100 m a.s.l. and 1.3–2.2 km from the village of Palazu Mic. The habitat consists of grazed steppe grasslands developed on Calcic Chernozems. Slopes range from gentle (3°) to steep (33°), with predominantly southern (S, SSE), eastern (ENE) and west-southwest (WSW) aspects. The soils are well-drained, with high calcium concentrations (3.41–5.41 mg/100 g soil), alkaline pH (8.03–8.20), moderate organic carbon contents (10.02–11.90%), and humus contents of 17.28–20.52%.

Mineral nutrient concentrations were low (NH_4_^+^ 0.001–0.003 mg/100 g soil, NO_2_^−^ 0.000–0.017 mg/100 g soil, and NO_3_^−^ 0.306–0.849 mg/100 g soil), indicating low mineral nitrogen availability. The site is characterised by high temperatures during the warm season (BIO10 = 21.78–21.93 °C), low annual precipitation (BIO12 = 431–435 mm), and low summer precipitation (BIO18 = 129–131 mm), reflecting a pronounced seasonal water deficit.

Population density ranged from 36 to 628 individuals per 100 m^2^, a total of 4,589 individuals were recorded. Of these, 566 individuals were flowering, corresponding to 12.3% of the recorded individuals. Flowering tended to be more frequent at higher elevations and on gentle slopes; for example, the highest flowering rate (60.49%) was recorded at 99.7 m a.s.l. and a slope of 7.2°, whereas no flowering individuals were observed on slopes steeper than 20°.

Plant height ranged from 14 to 99 cm (plot-level mean values of 55–57 cm), with most individuals falling between 40 and 65 cm. The number of capitula in anthesis ranged from 5 to 87 per plant, with maximum values recorded on gently sloping, southeastern-facing sites. The number of buds ranged from 9 to 64, while most individuals had 4–7 leaves per plant (minimum 2, maximum 16). The leaves were 5–20 cm long and 1–5 cm wide (**Figure 8**). The population is located within the Natura 2000 site ROSAC0215 Recifii Jurasici Cheia.

**Figure 8:**
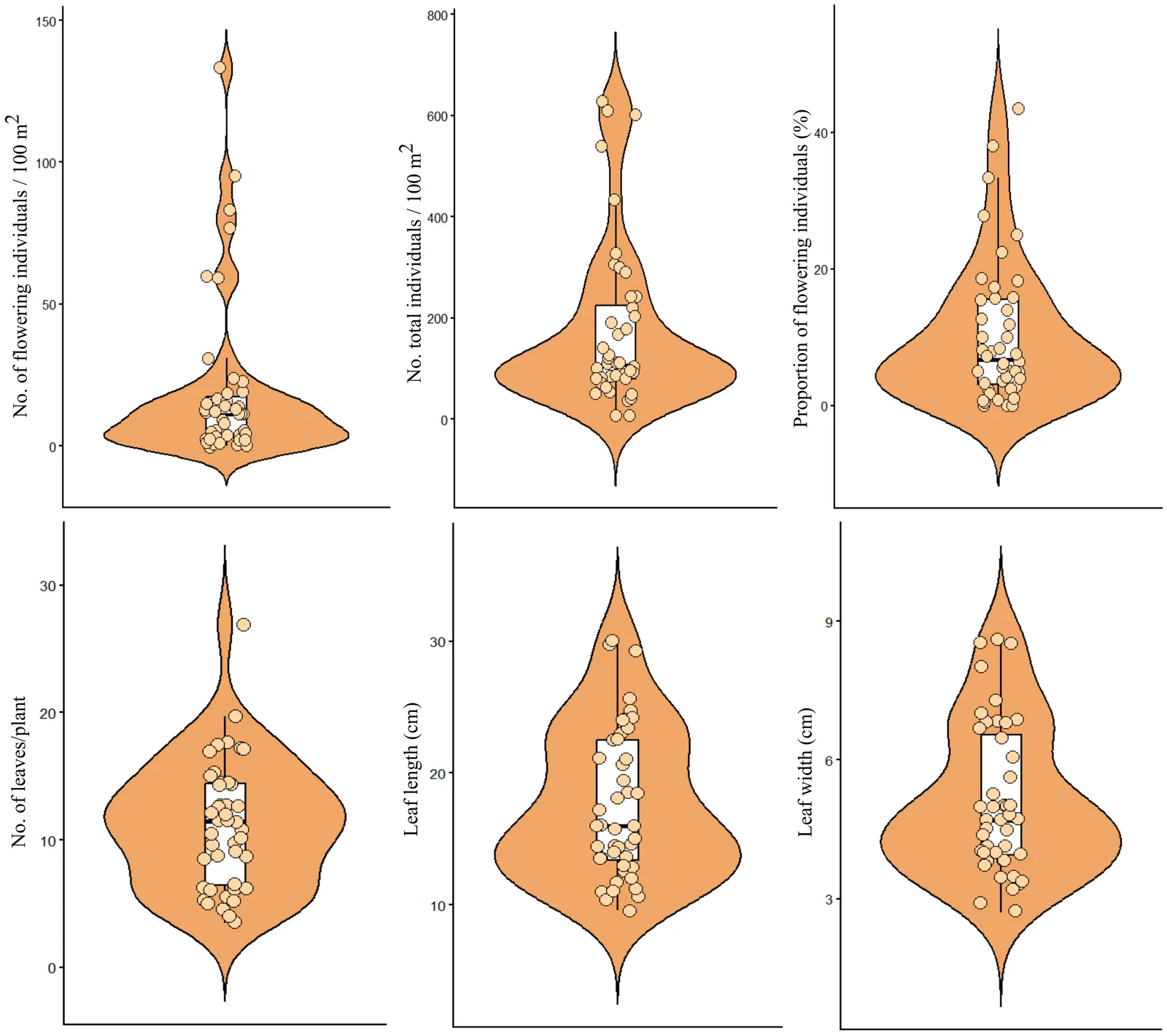
Distribution of demographic and morphological traits in *Centaurea jankae* based on sampled plots and individuals. Violin plots represent the distribution density of observations, embedded boxplots show the median and interquartile range, and points represent individual observations. Demographic variables (number of flowering individuals, total number of individuals, and proportion of flowering individuals) are expressed at the 100 m^2^ plot level, whereas leaf traits are based on individual plants.

##### 3.2.1.2 Slava Rusă

The population of *C. jankae* at Slava Rusă (Tulcea County, Romania) occurs at elevations of 105–109 m a.s.l., on gentle to moderate slopes (1.3–14.3°), with predominantly eastern to southeastern aspects (SE, SSE, ESE, and ENE). The soils are Calcic Chernozems with an alkaline reaction (pH = 7.89–8.14), variable organic carbon contents (6.1–11.9%) and moderate total phosphorus content of 1.015–1.693 mg/100 g soil. Nitrate nitrogen concentrations ranged from 0.483 to 0.771 mg/100 g soil. The grassland is currently non-grazed and shows low levels of anthropogenic disturbance.

Population density ranged from 85 to 167 individuals per 100 m^2^. A complete census recorded 1,533 individuals. The proportion of flowering individuals ranged from 3.57% to 25% across plots. A total of 210 flowering individuals were recorded, representing 13.7% of the population.

Plant height ranged from 57 to 145 cm (mean = 100 cm). The number of leaves per plant ranged from 7 to 51 (mean = 17), while leaf size showed moderate variation, ranging from 19.4 to 31 cm in length and 4–9 cm in width. The number of capitula in anthesis ranged from 1 to 40 per plant (mean = 14), and the number of buds varied between 1 and 25 (mean = 8) (**Figure 8**).

Individuals growing on eastern to southeastern aspects and on gentle slopes tended to be taller and to produce more leaves and flowering capitula. In contrast, plants growing on relatively steeper slopes (>12°) and northern or northwestern aspects were generally shorter and produced fewer leaves and capitula. The population occurs within the Natura 2000 site ROSCI0201 Podişul Nord Dobrogean.

##### 3.2.1.3 Jurilovca (Capul Doloşman)

The population of *C. jankae* at Capul Doloşman (Tulcea County, Romania) occurs at elevations of 21–30 m a.s.l., on Doloşman Hill. The habitat is sunny, with predominantly southern to southwestern aspects (S, SSE, SSW, SW, and WSW), and the slopes are very steep (40–54°). The soils are well-drained Calcic Chernozems with a strongly alkaline reaction and moderate nutrient contents.

Population density ranged from 51 to 306 individuals per 100 m^2^, and a complete census recorded 2,473 individuals. Higher population densities tended to occur on the less steep surfaces. The number of flowering individuals ranged from 0 to 133 per 100 m^2^. A total of 520 flowering individuals were recorded, representing 21% of all individuals.

In plots with comparatively gentler slopes, the proportion of flowering individuals frequently exceeded 30–40%. On very steep south-facing slopes, many plots contained only vegetative individuals. Plant height ranged from 70.7 to 103 cm. The number of leaves per plant varied between approximately 10.8 and 19.7, and leaves were 13.6–22.5 cm long and 4–6.8 cm wide. The number of capitula in anthesis remained low in many plots, ranging from 1 to 19 per plant (**Figure 8**).

The population occurs within the Natura 2000 site ROSCI0065 Danube Delta, in the xeric steppe habitat of the Capul Doloşman area.

##### 3.2.1.4 Nevsha (Taushan Tepe)

The population of *C. jankae* at Nevsha (Varna Province, Bulgaria) occurs approximately 1.5 km from the village of Nevsha, in non-grazed grasslands at elevations of 331–357 m a.s.l. Slopes range from 10° to 21°, and western and northwestern aspects predominate. The soils were classified as Calcic Chernozems and were well-drained, alkaline and characterised by moderate humus contents.

Population density ranged from 63 to 328 individuals per 100 m^2^, and a complete census recorded 3,211 individuals. The number of flowering individuals ranged from 1 to 133 per 100 m^2^, and a cumulative total of 1,511 flowering individuals was recorded across the surveyed plots, corresponding to 47.1% of the population. Plant height ranged from 36 to 115 cm, with most individuals between 50 and 70 cm. The number of leaves per plant ranged from 4 to 13, and the leaves were long and narrow (12–22 cm long and 4–5 cm wide; **Figure 8**).

A separate assessment conducted in 2023, two years after the 2021 survey referenced above, estimated the entire population at approximately 5,925 individuals, including 1,958 generative and 3,967 vegetative individuals, corresponding to 33.0% generative individuals. However, this estimate was not considered directly comparable with the 2025 plot-based counts because identical survey areas and census methods could not be confirmed.

##### 3.2.1.5 Ravna

The *C. jankae* population at Ravna (Varna Province, Bulgaria) shows a fragmented spatial distribution and strong variation in local density. The total population size was 1199 individuals. Population density ranged from 6 to 242 individuals per 100 m^2^. Most individuals occurred at elevations above 100 m a.s.l.

A total of 112 flowering individuals were recorded, representing 9.3% of all individuals. Most plots contained no flowering individuals. In plots where flowering individuals were present, their density was generally low (1–8 individuals per 100 m^2^).

The soils were classified as Calcic Chernozems. Southwestern aspects predominated, and slopes ranged from 5.9° to 21°. Mature individuals reached heights of 51–136 cm and had leaves 21–30 cm long and 7–8.6 cm wide (**Figure 8**). Only a small number of vigorous individuals were observed, mainly in lower-lying areas, whereas juvenile individuals occurred in more wind-exposed microsites.

The population showed a low flowering proportion and spatially uneven reproductive performance. However, its temporal trend could not be determined because comparable repeated censuses were unavailable. The observed population structure suggests the persistence of mature individuals but potentially limited recruitment. Long-term persistence may be influenced by local water availability, competition and disturbance regimes, although these relationships require further investigation.

##### 3.2.1.6 Venchan

The *C. jankae* population at Venchan (Varna Province, Bulgaria) was surveyed in a relatively homogeneous open habitat. The elevation ranged from 144 to 145 m a.s.l., with a west-southwestern aspect and a gentle slope (7.8°). A complete census recorded 1,469 individuals, of which 429 were flowering (29.2%). The proportion of flowering individuals was approximately 29%, indicating relatively high reproductive activity (**Figure 8**).

Grazing damage of inflorescences was observed, similar to that recorded at Palazu Mic (Constanţa County, Romania), and may reduce flowering and seed production. Flowering individuals occurred mainly in open areas, whereas vegetative individuals extended towards the edge and into a nearby pine plantation.

The population showed a relatively high local density and a substantial proportion of flowering individuals. However, grazing and shading associated with the pine plantation may locally limit reproductive performance and the availability of open habitat.

### 3.3 NMDS ordination

NMDS ordination revealed differences in floristic composition among the five *C. jankae* populations, with an acceptable bivariate solution (stress = 0.145) (**Figure 9**). PERMANOVA confirmed these differences (pseudo-F = 14.24; R^2^ = 0.548; p < 0.001), and multivariate dispersion did not vary significantly between groups (F = 1.72; p = 0.160), suggesting that the differences are due to composition itself, not to unequal heterogeneity within populations. All pairwise comparisons remained significant after Holm correction (**Supplementary Table 5**), although the weakest separation was between Nevsha and Ravna (R^2^ = 0.233; adjusted p = 0.0015).

**Figure 9:**
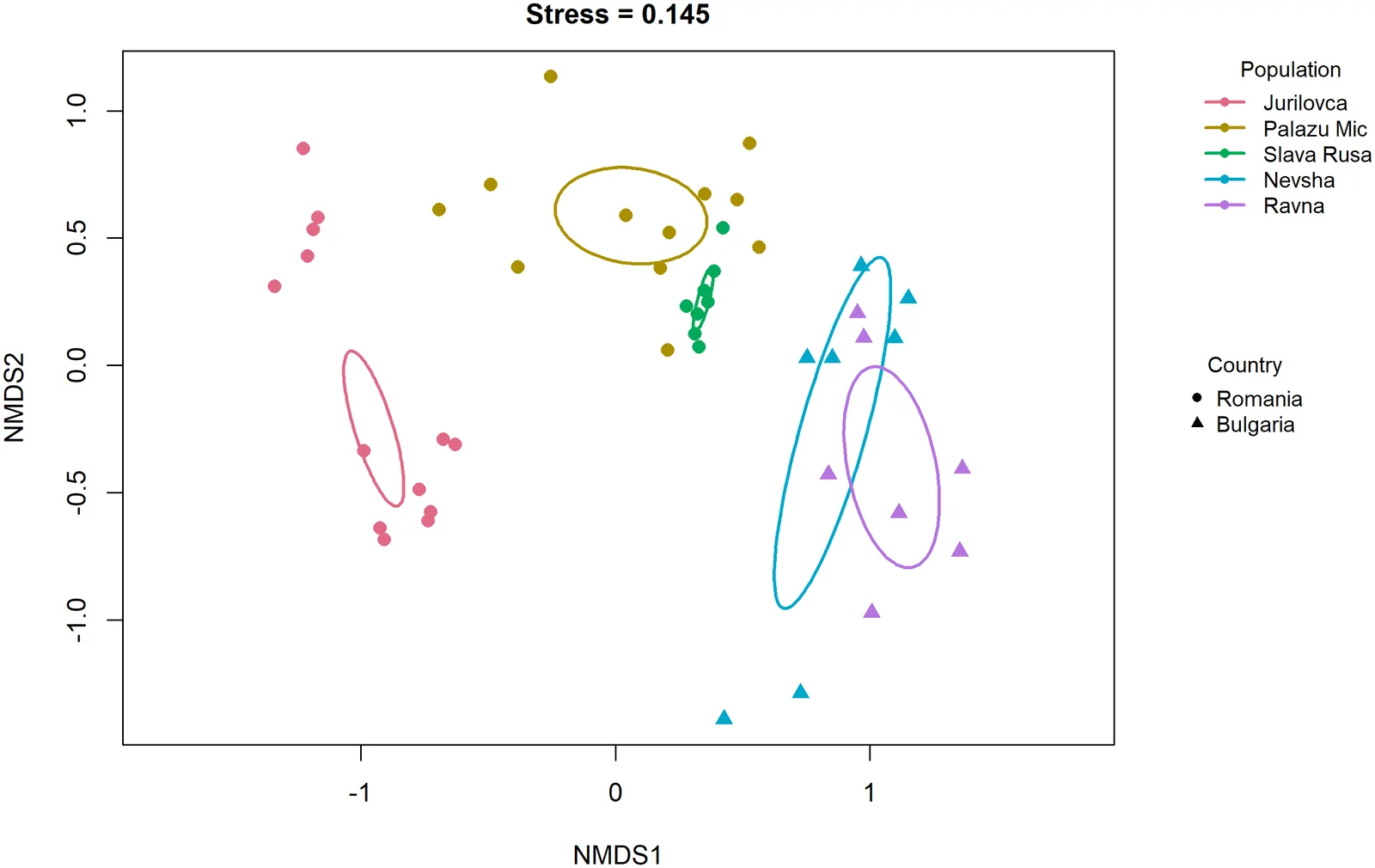
Non-metric multidimensional scaling ordination of vegetation composition in 52 phytocoenological relevés from five populations of *Centaurea jankae* in Romania and Bulgaria. The ordination was based on Bray–Curtis dissimilarities calculated from square-root-transformed species-cover values. Colours indicate populations, symbols indicate countries, and ellipses represent 95% confidence regions around group centroids. Stress = 0.145.

To check the robustness of the result, we repeated the analysis excluding the target species, *C. jankae*. The results were practically identical (stress = 0.148; pseudo-F = 14.21; R^2^ = 0.547; p < 0.001), a sign that the observed differentiation is not due solely to the variation in the abundance of this species.

### 3.4 Spatial modelling

#### 3.4.1 SDM performance

The MaxEnt model yielded a mean test AUC of 0.930 (± 0.056 SD) across five cross-validation replicates (**Figure 10**). At the 10-percentile training-presence threshold, the mean test omission rate was 0.14.

**Figure 10:**
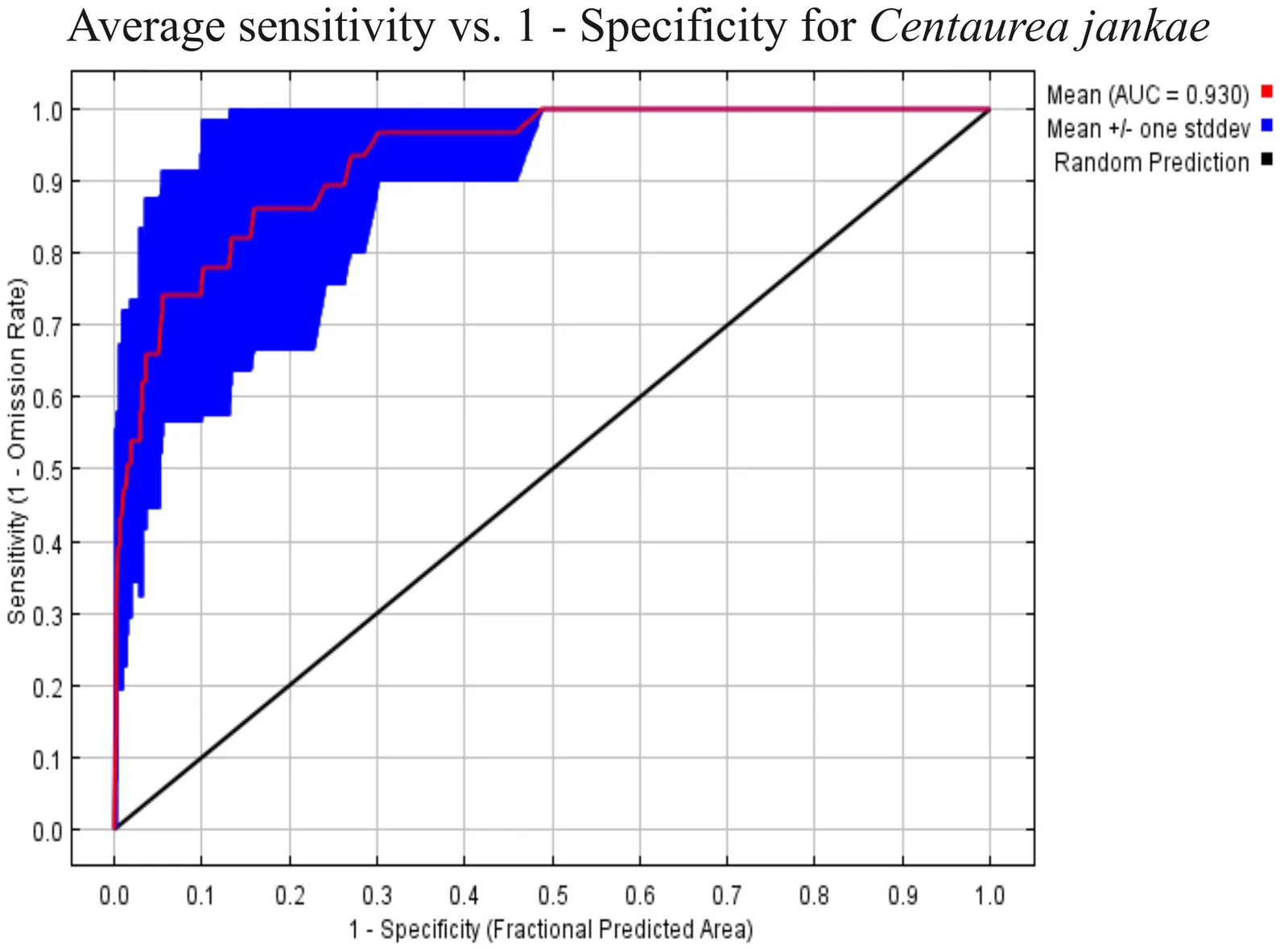
Receiver operating characteristic (ROC) curve for species distribution prediction.

#### 3.4.2 Current potential distribution

The MaxEnt model indicated that the highest values ​​of habitat suitability for *C. jankae* were concentrated in the central-northern part of the Dobrogea region. In Romania, most areas with a high probability of occurrence were located within the Natura 2000 site ROSAC0215 Recifii Jurasici Cheia, including Palazu Mic, Gura Dobrogei, Sitorman Quarry, Cheia and Târguşor (Constanţa County). Suitable habitats were also identified in the Natura 2000 site ROSCI0065 Danube Delta, in the Jurilovca–Capul Doloşman area (Tulcea County).

In Bulgaria, areas with high suitability were located within the Natura 2000 sites BG0000104 Provadiysko–Royaksko plato and BG0000623 Taushan tepe. Areas of high suitability largely coincide with limestone plateaus and south-facing slopes, which are characteristics of steppe and sub-Mediterranean habitats in Dobrogea (**Figure 3**).

The habitat suitability assessment indicated that 28,361.57 km^2^ (88.8%) of the study area was classified as unsuitable (habitat suitability < 0.2). Areas with low habitat suitability (0.2–0.4) covered 1,790.64 km^2^ (5.6%), medium habitat suitability (0.4–0.6) accounted for 746.73 km^2^ (2.3%), and high habitat suitability (≥ 0.6) occupied 1,025.31 km^2^ (3.2%) of the study area (**Figure 11**).

**Figure 11:**
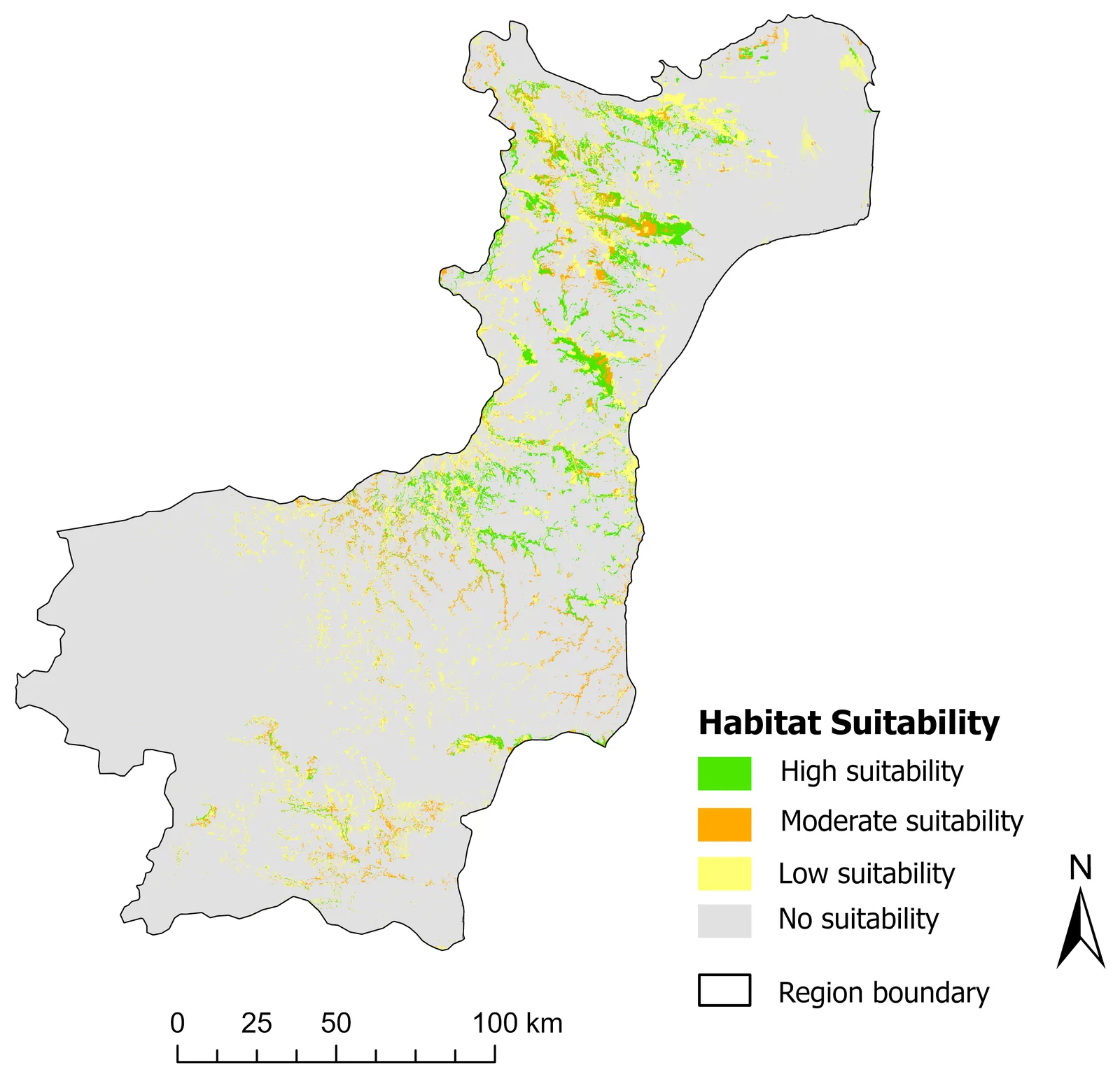
Spatial distribution of habitat suitability for *Centaurea jankae*.

#### 3.4.3 Analysis of variable contributions

Predictor importance was evaluated using both percent contribution and permutation importance provided by the MaxEnt model (**Table 3**).

**Table 3:** Variable contributions to the MaxEnt model for *Centaurea jankae*.

Variable	Percent contribution	Permutation importance
Land use (LULC)	42.8	39.4
Soil	24.3	4.5
Slope	14.9	12.1
Precipitation of the warmest quarter (BIO18)	12.9	31.9
Temperature annual range (BIO7)	5.1	12

LULC showed the highest percent contribution (42.8%) and permutation importance (39.4%) in the fitted model. Soil type accounted for 24.3% of percent contribution but showed substantially lower permutation importance (4.5%). High suitability values were associated with Leptosol, Greyzem, and Chernozem soils (**Figures 12A, B**). Slope showed a contribution (14.9%) and a permutation importance of 12.1% with the response curve indicating higher habitat suitability on slopes ranging from approximately 20° to 39° (corresponding to DN values of 195 to 230).

**Figure 12:**
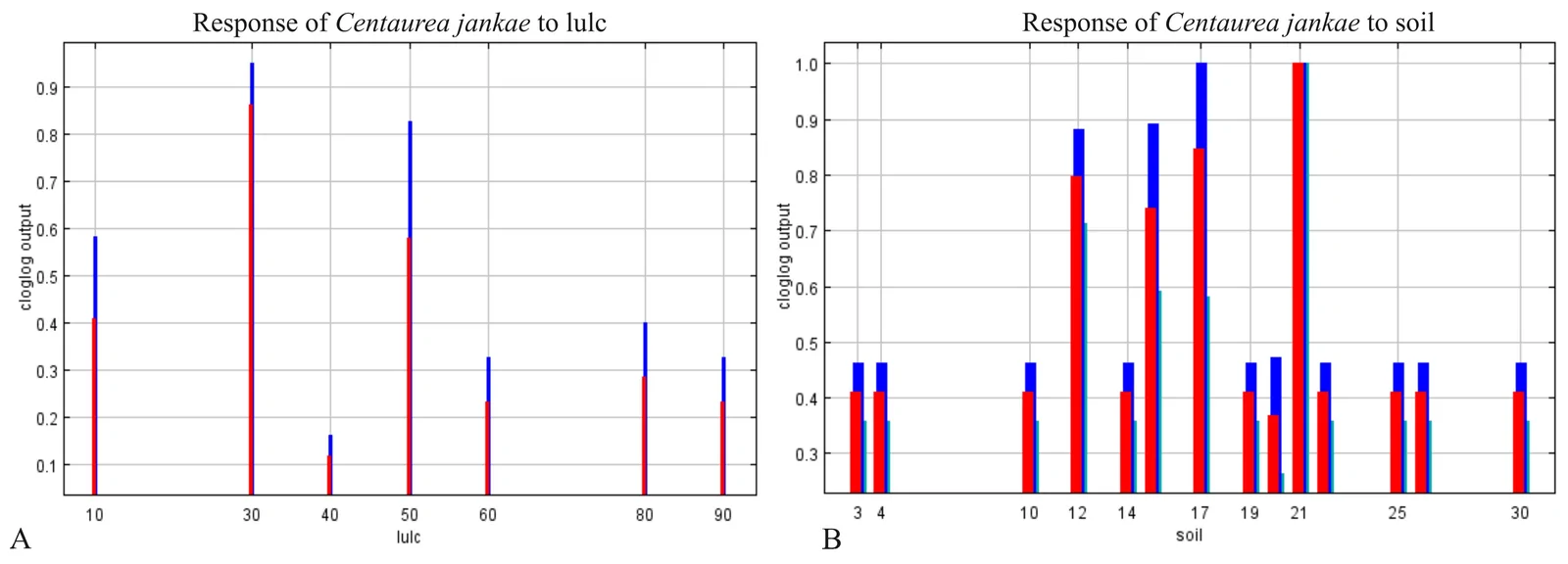
Response curves for the categorical variables of the SDM model: response of species to **(A)** land use/cover, **(B)** soil type. LULC: 10 – Tree cover; 30 – Grassland; 40 – Cropland; 50 – Built-up; 60 – Bare/Sparse vegetation; 80 – Permanent water bodies; 90 – Herbaceous wetland. Soil: 3 – Water body; 4 – Marsh; 10 – Arenosol; 12 – Chernozem; 14 – Cambisol; 15 – Fluvisol; 17 – Greyzem; 19 – Histosol; 20 – Kastanozem; 21 – Leptosol; 22 – Luvisol; 25 – Phaeozem; 26 – Planosol; 30 – Solonchak.

Among the climatic predictors, precipitation of the warmest quarter (BIO18) showed a relatively small contribution (12.9%) but a higher permutation importance (31.9%), indicating that this variable influenced model performance. The response curve showed a negative relationship between summer precipitation and habitat suitability. Predicted suitability was highest at relatively low precipitation values (approximately 100–120 mm) and decreased progressively as precipitation increased beyond 140–150 mm.

The temperature annual range (BIO7) had a low contribution (5.1%) and a permutation importance of 12%. The response curve suggested a tendency towards higher suitability at larger temperature ranges, with maximum values occurring at approximately 32–35 °C (**Figures 13A–C**).

**Figure 13:**
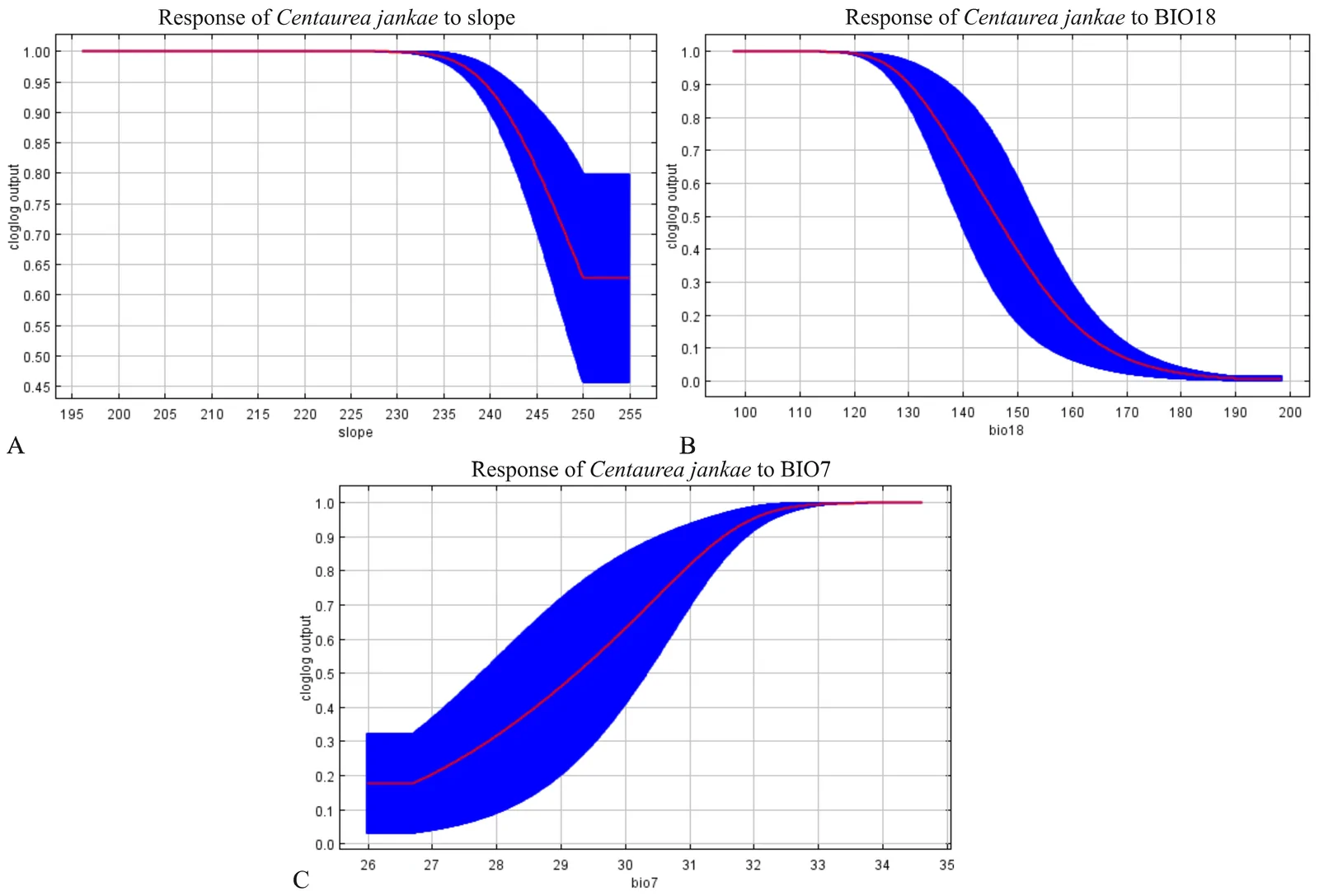
Response curves for the continuous variables of the SDM model: **(A)** response of the species to slope, **(B)** precipitation of the warmest quarter (BIO18), and **(C)** temperature annual range (BIO7; BIO5 − BIO6). The slope variable was derived from a raster in the SLOP format, in which pixel values are stored as Digital Numbers (DN) ranging from 0 to 250, rather than as angular degrees. For input into MaxEnt, the original DN values were used directly. These values can be converted to slope angles (in degrees) using the standard conversion function [Slope (°) = arccos (DN/250) × 180/π]. Consequently, in the response curve shown in panel **(a)**, the DN values on the x-axis correspond to the following slope angles: DN 195 ≈ 38.7°, DN 200 ≈ 36.9°, DN 210 ≈ 32.9°, DN 220 ≈ 28.4°, DN 230 ≈ 23.1°, DN 240 ≈ 16.3°, DN 245 ≈ 11.5°, DN 250 ≈ 0°.

Overall, predictor-importance rankings differed depending on the metric considered, although LULC showed consistently high values for both percent contribution and permutation importance.

### 3.5 Local conservation condition

The population-level assessment indicated a predominantly unfavourable conservation status: four of the six populations (Jurilovca–Capul Doloşman, Palazu Mic, Venchan, and Ravna) were classified as U1 (unfavourable–inadequate), and Slava Rusă and Nevsha were classified as XX (unknown), although their current status was assessed as favourable. The difference between the two groups is determined by the uncertainty regarding future evolution, not by the current status of the populations. Slava Rusă and Nevsha include large populations, habitats assessed as favourable (FV) and low current pressures, and no major pressures were identified for Slava Rusă. However, both populations were classified as XX in the overall local assessment, as future prospects and temporal trends could not be established. At Slava Rusă, population occupancy and trend have not been quantified, and at Nevsha, regional isolation and the risk of afforestation maintain a high level of uncertainty regarding future evolution. Therefore, the two populations are currently in a favourable state, but their future evolution cannot be established based on the available data, which justifies classification as XX, rather than FV.

The four populations classified in the U1 category presented different causes of the unfavourable status. At Jurilovca, the demographic status is still favourable (FV), but the small area and fragmentation of the habitat, together with uncertain future prospects, determine the overall unfavourable assessment. Palazu Mic and Ravna presented the most problematic elements, being assessed as U1 for almost all components, due to reduced reproduction, habitat degradation through grazing or drought and limited regeneration. At Venchan, the demographic status is favourable, but the habitat is affected by shading and grazing associated with the neighbouring pine plantation.

The identified pressures highlight a gradient of vulnerability, from Slava Rusă, where the main limitation is the restricted local distribution, to Ravna, where habitat fragmentation, interspecific competition, reduced regeneration and water scarcity accumulate. Recurrent anthropogenic pressures, in particular overgrazing and inflorescence damage, affect most populations and are accompanied by habitat-related threats, such as fragmentation, woody vegetation expansion and drought (**Table 4**).

**Table 4:** Population-level assessment of the local conservation condition of the six confirmed populations of *Centaurea jankae*.

Population	Population condition	Local occupancy and fragmentation	Habitat condition	Future prospects	Main pressures	Overall local condition
Palazu Mic	U1	U1	U1	U1	Grazing, damage to inflorescences and seasonal drought/water deficit	U1
Slava Rusă	FV	XX	FV	XX	No major current pressure observed; restricted local distribution	XX
Jurilovca–Capul Doloşman	FV	U1	U1	XX	Restricted habitat extent and fragmentation; agricultural, pastoral and archaeological pressures	U1
Nevsha	FV	FV	FV	XX	Restricted regional distribution and isolation; afforestation a potential threat	XX
Ravna	U1	U1	U1	U1	Habitat fragmentation, limited regeneration, competition and restricted water availability	U1
Venchan	FV	U1	U1	U1	Grazing of inflorescences and shading/encroachment from the pine plantation	U1

FV, favourable local condition; U1, unfavourable–inadequate local condition; U2, unfavourable–bad local condition; XX, unknown. Temporal trends were classified as XX because previous and current population estimates were not obtained using demonstrably comparable survey areas and census methods. The assessments represent population-level evaluations of local conservation condition and are not equivalent to formal Article 17 assessments conducted at the Member State biogeographical-region level. Detailed evidence supporting each assessment (individual counts, densities, flowering proportions, and full justifications) is provided in **Supplementary Table 6**.

Current population and habitat conditions were most favourable at Slava Rusă and Nevsha, where relatively large populations occurred in habitats with low levels of direct disturbance. In contrast, populations at Jurilovca–Capul Doloşman, Palazu Mic, Venchan, and Ravna presented unfavourable-inadequate local conditions, associated with habitat restriction or fragmentation, grazing, damage to inflorescences, shading, reduced flowering rates or limited regeneration. However, future prospects and temporal population trends remained uncertain for several populations, as comparable long-term monitoring data were not available.

## 4 Discussion

### 4.1 Research history

The first records of *Centaurea jankae* in Dobrogea date to the second half of the nineteenth century. Brândză (1884, 1898) reported the presence from the arid hills between Babadag Forest and the village of Caugagia (locus classicus) (Tulcea County). The species was formally described by D. Brândză based on material collected in Dobrogea.

Later, Prodan (1911, 1913, 1930, 1935, 1939, 1964) reported several populations in Dobrogea, including the arid hills between Babadag Forest and Caugagia, Jurilovca and Capul Doloşman and the area between Caugagia and Tariverde. During the same period, Petre Enculescu collected specimens in 1912 from the steppe area south of Babadag, on rocky skeletal soils.

In the first half of the twentieth century, Borza (1947) included *C. jankae* in the flora of central Dobrogea and emphasised its endemic character. The species was subsequently included in the national floristic synthesis of Prodan and Nyárády (1964).

During the second half of the twentieth century, studies by Dihoru and Doniţă (1970); Horeanu (1973), and Burduja and Horeanu (1976) expanded the known distribution of the species. They documented additional locations on the Casimcea Plateau (including Palazu Mic), Tariverde, Babadag Forest and other xerophilous areas of Dobrogea. Herbarium material collected by G. Dihoru also documents the species at Slava Rusă (Tulcea County). Later, Parincu (1997) and Parincu et al. (1998) reported its presence at Dumbrăveni, while an unpublished herbarium record by Negrean (2001) documented the species at Cotu Văii (**Supplementary Table 1**).

In Bulgaria, *C. jankae* was reported relatively late compared to the Romanian records. Petrova (2007) documented the presence of the species in northeastern Bulgaria at Taushan Tepe (Varna district), and later a second locality was confirmed at Pobiti Kamani (Venchanski Dyuz). Subsequent surveys reassessed the populations known as Taushan Tepe and Probitiya Kamak and confirmed the highly restricted and fragmented distribution of the species in Bulgaria (Nikolov and Bancheva, 2021). Petrova’s study reported the species from sites near Nevsha and Venchan, while the later Bulgarian assessment referred to Taushan Tepe and Probitiya Kamak.

Other floristic syntheses and regional studies (Dihoru and Pîrvu, 1987; Ciocârlan and Sârbu, 1998; Oţel, 2000; Negrean and Anastasiu, 2003; Oprea, 2005; Sârbu et al., 2013) confirmed the restricted distribution of C. jankae in Dobrogea. The principal historical locations were situated around Jurilovca–Capul Doloşman, Babadag Forest, the Casimcea Plateau, Palazu Mic, Tariverde and Dumbrăveni.

The present field investigations showed that several historically reported locations could not be reconfirmed. In particular, the species was not found at Cheile Dobrogei or Cotu Văii during the 2023–2025 surveys, despite earlier observations. This may reflect local decline, very small population size or uncertainty regarding the precise position of some historical records; therefore, local extinction cannot be inferred from the available evidence. In contrast, the populations at Palazu Mic and Jurilovca–Capul Doloşman were reconfirmed. These findings support the conclusion that the current known distribution of *C. jankae* in Dobrogea is highly fragmented.

### 4.2 Population size and reproductive structure

Comparisons between the current and previously published estimates of *C. jankae* population size and reproductive structure should be interpreted cautiously because survey extent and census methods were not consistently documented or directly comparable. For the Bulgarian populations, previous studies provide useful reference values (Petrova, 2007; Nikolov and Bancheva, 2021). At Nevsha (Taushan Tepe), generative individuals represented approximately 29.7% of the population in 2021 (Nikolov and Bancheva, 2021), whereas flowering individuals represented 47.1% of the individuals recorded in the present study. The population from Venchan (Probitiya Kamak) showed a flowering proportion broadly comparable to the generative proportion reported in 2021 (approximately 24.2%; Nikolov and Bancheva, 2021), without implying a temporal trend, while the population from Ravna remains dominated by vegetative individuals, confirming previous observations (Petrova, 2007).

For Romania, the available literature provides lower historical estimates, although these values are not directly comparable with the present data. According to Anastasiu and Negrean (2008), the populations at Cotu Văii and Palazu Mic previously included approximately 60 individuals, while subsequent field checks at Palazu Mic failed to identify any individuals, suggesting local extinction. In contrast, the largest population was reported at Jurilovca (Capul Doloşman), with approximately 300 individuals (Anastasiu and Negrean 2008). Similarly, Sârbu et al. (2006) describe the population at Jurilovca (Capul Doloşman) as the densest, with almost 300 individuals, most of them flowering. More recent data from the Management Plan of the North Dobrogean Plateau (2024) indicate that the population of *C. jankae* at Slava Rusă consists of approximately 450 individuals on an area of 500 m^2^, corresponding to a density of approximately 90 individuals per 100 m^2^.

Complete population censuses recorded 4,589 individuals at Palazu Mic, 2,473 at Jurilovca–Capul Doloșman, and 1,533 at Slava Rusă. However, these higher values cannot be interpreted as population increases because the historical and current assessments differed in survey area and census design. Flowering individuals represented 12.3% of the recorded individuals at Palazu Mic, 13.7% at Slava Rusă and 21.0% at Jurilovca–Capul Doloşman. Consequently, the available data describe current differences in population and reproductive structure, but do not support temporal trend classification.

### 4.3 Comparison with previous conservation assessments

The results obtained in this study are broadly comparable with previous Article 17 assessments, although the two evaluations operate at different spatial scales and are not directly equivalent. In Romania, *C. jankae* was assessed as Unfavourable-Inadequate (U1) during the 2007–2012 (European Environment Agency, 2007-2012) and 2013–2018 (European Environment Agency, 2013-2018). The current local assessment identified unfavourable–inadequate conditions at Jurilovca–Capul Doloşman and Palazu Mic. At Slava Rusă, current population and habitat conditions were favourable, but the overall local condition was classified as XX because temporal trend and future prospects remained unknown.

In Bulgaria, *C. jankae* was previously assessed as Favourable (FV) at the biogeographical region level. However, field data (2021) indicate variation among individual populations. In the present study, Venchan and Ravna were classified as U1. At Nevsha, current population and habitat conditions were favourable, but the overall local condition remained XX because comparable temporal data and reliable future-prospect information were unavailable.

### 4.4 Spatial modelling

The limited potential distribution is consistent with the endemic and specialised character of *C. jankae*, described in the literature (Petrova and Vladimirov, 2010; Sârbu et al., 2013) as a species strictly related to calciphilic steppe grasslands in Dobrogea. The association with communities of the *Festuco*-*Brometea* class is consistent with vegetation studies in Europe (Dengler et al., 2012; Mucina et al., 2016), which highlight the fragmented and insular character of calcareous grasslands in southeastern Europe. The spatial fragmentation reflects the discontinuous distribution of calcareous outcrops, a factor recognised as a major determinant of the distribution of calciphilic species in karst and loess-calcareous regions (Dengler et al., 2012). The relatively high contribution of land use/land cover is consistent with an association between predicted habitat suitability and land-cover characteristics; however, this model-based association should not be interpreted as evidence of a direct causal limitation. Studies (Guisan and Thuiller, 2005; Franklin and Miller, 2009) on modelling the distribution of species in semi-natural habitats show that land use variables can outperform climate variables in explaining current distribution, especially for species with specialised habitats.

In south-eastern Europe, the loss and degradation of semi-natural grasslands through abandonment, afforestation or agricultural conversion are recognised as major drivers of decline for steppe species (Veen et al., 2009; European Commission, 2019). In this context, the model results are consistent with the literature indicating land-use changes as the main determinant of the current distribution in calcareous grasslands.

The association with calcareous soil groups, particularly Leptosols and Chernozems, is consistent with the calciphilic ecology of the species. Edaphic specialization is frequently associated with restricted areas and fragmented distributions, as calcareous soils appear as islands in the landscape (Pärtel et al., 2007; Dengler et al., 2012). Studies on European calciphilic species show that the substrate can represent a stronger barrier than climate in determining distribution (Pärtel, 2002).

The negative relationship between habitat suitability and precipitation in the warmest quarter (BIO18) indicates that, within the fitted model, lower summer precipitation was associated with higher predicted suitability for *C. jankae*. Experimental studies in temperate and semi-arid grasslands show that altered precipitation can affect plant physiological performance and, depending on ecosystem context, community composition (Song et al., 2016; Yang et al., 2019).

However, these studies do not establish the mechanism underlying the response of *C. jankae*. The BIO18 response should therefore be interpreted as a model-based environmental association rather than evidence that higher summer precipitation directly reduces the species through increased competition; testing such a mechanism would require targeted field or experimental data.

The MaxEnt model estimates the current potential distribution of *C. jankae*, but several limitations should be considered. It was calibrated using 27 spatially thinned records from six geographically clustered populations. MaxEnt can perform adequately with small samples when predictors are filtered and model complexity is constrained (Hernandez et al., 2006; Pearson et al., 2007), but the small number of records limits the interpretation of the fitted response curves. Model performance was evaluated by random cross-validation rather than by a spatially independent partitioning scheme. Because the occurrence records are spatially clustered, autocorrelation between training and test folds may inflate the test AUC (0.930), which should therefore be interpreted cautiously. The interpretation of AUC in presence-background models also has limitations because its maximum attainable value may be below 1. Percent contribution and permutation importance also differed for some predictors, particularly soil type, so these values should not be treated as exact measures of each variable’s effect. Additional occurrence records, spatial block cross-validation and independent field validation would improve model evaluation. More generally, SDMs describe potential suitability rather than actual distribution because they do not explicitly account for demographic processes, dispersal limitations or biotic interactions (Elith and Leathwick, 2009). Differences between predicted and observed distributions can therefore occur (Tsiftsis et al., 2024), particularly when dispersal, habitat fragmentation or other ecological barriers limit species occurrence (Svenning and Skov, 2004; Beale and Lennon, 2012).

An additional limitation is that possible hybridisation was not assessed in the present study. Hybridisation and introgression are well documented within *Centaurea* sect. *Acrocentron*, including its Balkan lineages (Font et al., 2002, 2009; Novaković et al., 2022). Therefore, undetected hybridisation cannot be ruled out as a potential source of variation in the morphological and ecological patterns observed here. Population-level molecular analyses would be required to evaluate this possibility specifically for *C. jankae*.

Dry calcareous grasslands, such as those inhabited by *C. jankae*, are among the most species-rich and priority habitats for conservation in Europe. Unfortunately, these grasslands are strongly threatened by land-use change, abandonment and fragmentation (European Commission, 2019). In Dobrogea, calcareous steppe grasslands have been shown to have particular floristic importance but now occur in isolated fragments, mostly in protected areas (Sârbu et al., 2009). The dynamics of these habitats are shaped not only by contemporary environmental constraints but also by historical legacies of land use, which influence both succession patterns and biodiversity (Labadessa et al., 2023).

Overall, H1 was supported, as the currently confirmed populations were geographically isolated and consistently associated with fragmented open calcareous steppe habitats. H2 received partial support: population structure and reproductive performance varied among populations in association with local environmental conditions and observed pressures, although these relationships were not tested causally. H3 was supported, as habitat suitability was strongly associated with land-cover and substrate characteristics, with additional contributions from topographic and climatic variables, and highly suitable habitat occupied only a small proportion of the study area.

## 5 Conclusions

Of the 14 historically reported populations of *C. jankae* in Romania and Bulgaria, six were confirmed during the 2023–2025 surveys: three in Romania and three in Bulgaria. The confirmed populations differed in abundance, flowering proportion, morphological traits and vegetation composition, while remaining consistently associated with fragmented, open calcareous steppe habitats. In the fitted MaxEnt model, land use/land cover showed the highest percent contribution, whereas soil type also contributed to predicted habitat suitability. Areas classified as highly suitable represented 3.2% of the study area.

The SDM results should be interpreted cautiously because model calibration was based on only 27 spatially filtered presence records clustered within six populations and model evaluation did not use a spatially independent validation scheme. In addition, interpretation of population trends is limited by the absence of fully comparable historical censuses.

At the population level, four populations were assessed as having an unfavourable–inadequate conservation condition (U1), whereas the overall condition of two populations remained unknown (XX) because temporal trends and future prospects could not be assessed reliably. Conservation measures should therefore prioritise standardised long-term demographic monitoring, the maintenance of open calcareous grasslands, the prevention of excessive grazing and woody encroachment, and population-specific management.

Overall, the results supported H1 and H3, whereas H2 received partial support because the observed relationships between population performance and local pressures were descriptive rather than experimentally or statistically demonstrated.

## Data Availability

The original contributions presented in the study are includedin the article/**Supplementary Material**. Further inquiries can bedirected to the first author SDC simonachirilasc@yahoo.com.
