# Supplementary material for: Updated chorology, habitat ecology, conservation assessment and habitat suitability of Centaurea jankae in Romania and Bulgaria
Source: Front Plant Sci. 2026 Sep 18;17:1904573. doi: 10.3389/fpls.2026.1904573 (PMC13631827; doi:10.3389/fpls.2026.1904573)
Supplement: Supplementary file 1 [file SupplementaryFile1.zip › Supplementary Table S6.docx]

**Supplementary Table S6.** Detailed evidence supporting the population-level assessment of local conservation condition in the six confirmed populations of *Centaurea jankae.*

| **Assessment component** | **Slava Rusă** | **Jurilovca–Capul Doloșman** | **Palazu Mic** | **Nevsha** | **Venchan** | **Ravna** |
| --- | --- | --- | --- | --- | --- | --- |
| **Population evidence, 2025** | 1,533 individuals; density 85–167 individuals/100 m²; 210 flowering individuals (13.7%); complete population census; temporal trend XX | 2,473 individuals; density 51–306 individuals/100 m²; 520 flowering individuals (21.0%); trend XX | 4,589 individuals; density 36–628 individuals/100 m²; 566 flowering individuals (12.3%); trend XX | 3,211 individuals; density 63–328 individuals/100 m²; 1,511 flowering individuals (47.1%); trend XX | 1,469 individuals recorded across the surveyed plots; density 15–155 individuals/100 m²; 429 flowering individuals (29.2%); temporal trend XX | 1,199 individuals; density 6–242 individuals/100 m²; 112 flowering individuals (9.3%); trend XX |
| **Population condition** | FV — relatively large population, moderate density and reproductive individuals present | FV — relatively large current population, with flowering individuals recorded, although several very steep plots contained only vegetative individuals | U1 — high abundance but spatially constrained reproductive performance, with no flowering individuals recorded in plots on slopes steeper than 20° | FV — a large population persists in several groups at Taushan Tepe; current occupancy is maintained, but temporal change in occupied area was not quantified | FV — high local density and a substantial proportion of flowering individuals | U1 — low flowering proportion, absence of flowering individuals from most plots and evidence of limited regeneration |
| **Local occupancy and spatial fragmentation** | XX — a single confirmed population occurs within ROSCI0201; the current occupied area and its temporal trend were not quantified | U1 — the population is restricted to the Capul Doloșman steppe habitat and is isolated within a strongly fragmented regional distribution | U1 — the population consists of spatially separated nuclei in a region where several historical occurrences were not reconfirmed | FV for current occupancy; temporal trend XX — a large population persists in several groups at Taushan Tepe, but change in occupied area was not measured | U1 — the population is locally restricted, and vegetative individuals extend towards the margin and interior of a nearby pine plantation | U1 — explicitly fragmented spatial distribution |
| **Habitat condition** | FV — non-grazed steppe grassland with low levels of anthropogenic disturbance and apparently suitable edaphic conditions | U1 — suitable xeric calcareous habitat persists, but its local extent is restricted and much of the population occurs on very steep slopes | U1 — grazed steppe grassland exposed to pronounced seasonal water deficit | FV — non-grazed, well-drained calcareous grassland with no major current disturbance reported | U1 — open habitat remains suitable, but local shading and grazing affect parts of the population | U1 — habitat supports mature and juvenile individuals, but population performance varies strongly among microsites |
| **Main pressures** | No major current pressure was observed; restricted local distribution | Restricted habitat extent and fragmentation; agricultural and pastoral activities and archaeological works have been identified in the literature as potential local pressures | Grazing, observed damage to inflorescences and seasonal drought/water deficit | Restricted regional distribution and isolation; afforestation represents a potential threat documented for Bulgarian populations | Grazing of inflorescences and shading/encroachment associated with the pine plantation | Habitat fragmentation, limited regeneration, competition and restricted water availability |
| **Future prospects** | XX — the effects of future pressures and conservation measures were not quantitatively assessed | XX — future effects of the identified pressures were not quantified | U1 — continuation of grazing and water stress may reduce reproductive performance | XX — local future conditions were not assessed quantitatively | U1 — continued grazing and increasing shading may reduce flowering and available open habitat | U1 — persistence may be affected by low reproductive performance and limited regeneration if current conditions continue |
| **Confidence** | Medium for current population and habitat condition; low for temporal trend and future prospects | Medium for current demographic condition; medium–low for habitat trajectory and future prospects | Medium | Medium for current condition; low for temporal trend and future prospects | Medium | Medium |
| **Overall local condition** | XX; current local condition favourable | U1 | U1 | XX; current local condition favourable | U1 | U1 |

*FV, favourable local condition; U1, unfavourable–inadequate local condition; U2, unfavourable–bad local condition; XX, unknown. Temporal trends were classified as XX because previous and current population estimates were not obtained using demonstrably comparable survey areas and census methods. The assessments represent population-level evaluations of local conservation condition and are not equivalent to formal Article 17 assessments conducted at the Member State biogeographical-region level.*
