# Supplementary material for: Updated chorology, habitat ecology, conservation assessment and habitat suitability of Centaurea jankae in Romania and Bulgaria
Source: Front Plant Sci. 2026 Sep 18;17:1904573. doi: 10.3389/fpls.2026.1904573 (PMC13631827; doi:10.3389/fpls.2026.1904573)
Supplement: Supplementary file 1 [file SupplementaryFile1.zip › Supplementary Table S1.docx]

**Supplementary Table S1.** Herbarium records documenting historical occurrences of *Centaurea jankae* in Romania

Enculescu P., 11 July 1912, south of Babadag, rocky slopes with skeletal soil, BUC 264820–264822.

Negrean G., 3 June 2001, south of Cotu Văii, BUC, accession number unavailable.

Prodan I., 25 June 1911, between Babadag Forest and Caugagia, BUC 264997.

Prodan I., 1964, between Caugagia and Tariverde, H. Prodan herbarium, accession number unavailable.

Negrean G. & Constantinescu O., 1978, Capul Doloșman, BUCA 80811; HMR 2753.

Dihoru G., 2009, Slava Rusă Quarry, BUCA 150232.

Negrean G. & Constantinescu O., 1981, Capul Doloșman, herbarium record; accession number unavailable.

Negrean G., 1978, Capul Doloșman, herbarium record; accession number unavailable.
