# Supplementary material for: Updated chorology, habitat ecology, conservation assessment and habitat suitability of Centaurea jankae in Romania and Bulgaria
Source: Front Plant Sci. 2026 Sep 18;17:1904573. doi: 10.3389/fpls.2026.1904573 (PMC13631827; doi:10.3389/fpls.2026.1904573)
Supplement: Supplementary file 1 [file SupplementaryFile1.zip › Supplementary Table S2.docx]

**Supplementary Table S2.** Species distribution modelling (SDM) of *Centaurea jankae*

**Spatial modelling**

***Occurrence data and spatial thinning***

**Table A1.** Occurrence records retained after 100 m spatial thinning in R (version 4.5.0) using the *spThin* package and used for MaxEnt modelling.

| **No.** | **Species** | **Latitude** | **Longitude** |
| --- | --- | --- | --- |
| 1 | *Centaurea jankae* | 44.44344 | 28.51466 |
| 2 | *Centaurea jankae* | 43.92981 | 28.00782 |
| 3 | *Centaurea jankae* | 43.8052 | 28.33889 |
| 4 | *Centaurea jankae* | 44.81027 | 28.65976 |
| 5 | *Centaurea jankae* | 44.76242 | 28.93537 |
| 6 | *Centaurea jankae* | 44.81198 | 28.69272 |
| 7 | *Centaurea jankae* | 44.83425 | 28.53726 |
| 8 | *Centaurea jankae* | 44.86609 | 28.59878 |
| 9 | *Centaurea jankae* | 44.9218 | 28.26061 |
| 10 | *Centaurea jankae* | 44.57289 | 28.58711 |
| 11 | *Centaurea jankae* | 43.29496 | 27.30778 |
| 12 | *Centaurea jankae* | 43.24018 | 27.33974 |
| 13 | *Centaurea jankae* | 43.26401 | 27.39649 |
| 14 | *Centaurea jankae* | 44.49705 | 28.43682 |
| 15 | *Centaurea jankae* | 44.44426 | 28.51772 |
| 16 | *Centaurea jankae* | 44.44295 | 28.50495 |
| 17 | *Centaurea jankae* | 44.44242 | 28.50382 |
| 18 | *Centaurea jankae* | 44.44064 | 28.50993 |
| 19 | *Centaurea jankae* | 44.43734 | 28.51313 |
| 20 | *Centaurea jankae* | 44.43668 | 28.51413 |
| 21 | *Centaurea jankae* | 44.4431 | 28.51365 |
| 22 | *Centaurea jankae* | 44.76291 | 28.93456 |
| 23 | *Centaurea jankae* | 44.44095 | 28.51078 |
| 24 | *Centaurea jankae* | 44.4435 | 28.51284 |
| 25 | *Centaurea jankae* | 43.2384 | 27.33833 |
| 26 | *Centaurea jankae* | 43.29193 | 27.31108 |
| 27 | *Centaurea jankae* | 44.76347 | 28.93377 |

***Selection of environmental variables***

Environmental variable selection consisted of two sequential steps. Initially, an SDM was developed in MaxEnt using all 26 candidate environmental variables. Subsequently, Pearson's correlation coefficients were calculated in R for the continuous predictors, and a threshold of |r| > 0.7 was applied to identify highly correlated variables prior to selecting the final predictor set.

The SDM model was calibrated using the 27 occurrence records retained after spatial thinning at a minimum distance of 100 m. Only linear and quadratic feature classes were enabled, while all other MaxEnt settings were left at their default values. This preliminary model was used to provide an initial assessment of the relative importance and contribution of the environmental predictors, thereby supporting the subsequent variable selection process.

The results of this preliminary model, including the ROC curve, jackknife analysis, percentage contribution, permutation importance and model performance, as reported in the MaxEnt HTML output file, are presented in the following section.

**Figure A1.** The following picture shows the omission rate and predicted area as a function of the cumulative threshold. The omission rate is is calculated both on the training presence records, and (if test data are used) on the test records. The omission rate should be close to the predicted omission, because of the definition of the cumulative threshold.

**Figure A2.** The next picture is the receiver operating characteristic (ROC) curve for the same data. Note that the specificity is defined using predicted area, rather than true commission. This implies that the maximum achievable AUC is less than 1. If test data is drawn from the Maxent distribution itself, then the maximum possible test AUC would be 0.918 rather than 1; in practice the test AUC may exceed this bound.

                         

**Figure A3.** Response curves. These curves show how each environmental variable affects the Maxent prediction. The curves show how the predicted probability of presence changes as each environmental variable is varied, keeping all other environmental variables at their average sample value. In other words, the curves show the marginal effect of changing exactly one variable, whereas the model may take advantage of sets of variables changing together.

**Table A2.** Percentage contribution and permutation importance of the 26 candidate environmental variables in the initial MaxEnt model. Variable contribution (%) represents the relative contribution of each predictor during model training, whereas permutation importance (%) reflects the decrease in model performance after random permutation of the values of each variable. Values are presented as averages across replicate model runs.

| **Variable** | **Percent contribution** | **Permutation importance** |
| --- | --- | --- |
| lulc | 41.8 | 20.6 |
| soil | 22.4 | 2.6 |
| slope | 15.2 | 3.5 |
| bio16 | 6.3 | 0 |
| bio5 | 4.5 | 0 |
| bio18 | 4 | 18.8 |
| bio11 | 1.4 | 16.8 |
| bio7 | 1.4 | 21.4 |
| ph | 1.1 | 0.2 |
| bio2 | 0.4 | 0 |
| elevation | 0.4 | 5.9 |
| bio9 | 0.4 | 0.5 |
| bio6 | 0.3 | 6.7 |
| aspect | 0.2 | 0 |
| bio15 | 0.1 | 0 |
| bio13 | 0 | 2.8 |
| bio17 | 0 | 0 |
| bio8 | 0 | 0 |
| bio14 | 0 | 0 |
| bio19 | 0 | 0 |
| bio4 | 0 | 0 |
| bio12 | 0 | 0 |
| soc | 0 | 0 |
| bio10 | 0 | 0 |
| bio1 | 0 | 0 |
| bio3 | 0 | 0 |

**Figure A4.** The following picture shows the results of the jackknife test of variable importance. The environmental variable with highest gain when used in isolation is lulc, which therefore appears to have the most useful information by itself. The environmental variable that decreases the gain the most when it is omitted is lulc, which therefore appears to have the most information that isn't present in the other variables.

In the second step of the variable selection process, pairwise Pearson correlation coefficients were calculated in R for the continuous environmental predictors using a threshold of to identify highly correlated variables. The tables below present the environmental data used for the correlation analysis and the resulting Pearson correlation matrix.

**Table A3.** Environmental predictor values extracted at the occurrence records of *Centaurea jankae* used for Pearson correlation and VIF analyses.

| **BIO1** | **BIO2** | **BIO3** | **BIO4** | **BIO5** | **BIO6** | **BIO7** | **BIO8** | **BIO9** | **BIO10** | **BIO11** | **BIO12** | **BIO13** | **BIO14** | **BIO15** | **BIO16** | **BIO17** | **BIO18** | **BIO19** | **ELEVATION** | **SLOPE** | **ASPECT** | **PH** | **SOC** |
| --- | --- | --- | --- | --- | --- | --- | --- | --- | --- | --- | --- | --- | --- | --- | --- | --- | --- | --- | --- | --- | --- | --- | --- |
| 11.1875 | 9.074999809 | 28.62779999 | 854.9539795 | 28.20000076 | -3.5 | 31.70000076 | 19.61669922 | 0.766667008 | 21.7833004 | 0.766667008 | 435 | 54 | 24 | 22.52260017 | 136 | 80 | 131 | 80 | 93.22000122 | 249 | 53 | 76 | 331 |
| 11.44579983 | 9.024999619 | 28.92630005 | 841.1370239 | 28.20000076 | -3 | 31.20000076 | 15.7833004 | 2.450000048 | 21.79999924 | 1.049999952 | 470 | 53 | 29 | 16.94650078 | 138 | 93 | 132 | 94 | 119.8899994 | 245 | 225 | 72 | 363 |
| 11.69999981 | 8.883330345 | 29.1257 | 814.5050049 | 28 | -2.5 | 30.5 | 19.5333004 | 2.849999905 | 21.79999924 | 1.783329964 | 467 | 52 | 31 | 16.03969955 | 131 | 95 | 128 | 99 | 78.05000305 | 249 | 30 | 72 | 537 |
| 10.93330002 | 8.899999619 | 28.52560043 | 849.7230225 | 27.5 | -3.700000048 | 31.20000076 | 19.43330002 | 0.533333004 | 21.45000076 | 0.533333004 | 432 | 53 | 24 | 21.71360016 | 136 | 81 | 131 | 81 | 57.63999939 | 250 | 105 | 78 | 305 |
| 11.54170036 | 7.400000095 | 24.26230049 | 869.1339722 | 27.60000038 | -2.900000095 | 30.5 | 20.10000038 | 1.016669989 | 22.38330078 | 1.016669989 | 394 | 47 | 23 | 19.59889984 | 119 | 77 | 117 | 77 | -3 | 202 | 9 | 0 | 0 |
| 10.82079983 | 8.774999619 | 28.30649948 | 846.3220215 | 27.20000076 | -3.799999952 | 31 | 19.26670074 | 1.716670036 | 21.29999924 | 0.483332992 | 428 | 52 | 24 | 20.87849998 | 133 | 82 | 129 | 82 | 88.25 | 249 | 98 | 76 | 293 |
| 10.33749962 | 8.391670227 | 27.51370049 | 840.1279907 | 26.5 | -4 | 30.5 | 18.79999924 | 1.25 | 20.70000076 | 0.033333302 | 441 | 54 | 25 | 22.42429924 | 141 | 84 | 134 | 84 | 105.1100006 | 244 | 132 | 70 | 453 |
| 10.74170017 | 8.833330154 | 28.40299988 | 847.0560303 | 27.20000076 | -3.900000095 | 31.10000038 | 19.29999924 | 1.600000024 | 21.20000076 | 0.349999994 | 435 | 53 | 24 | 22.22979927 | 138 | 82 | 132 | 82 | 110.7399979 | 249 | 176 | 69 | 496 |
| 10.42080021 | 9.324999809 | 28.51679993 | 890.1359863 | 28 | -4.699999809 | 32.70000076 | 19.5 | -0.56666702 | 21.33329964 | -0.56666702 | 456 | 59 | 25 | 25.35980034 | 151 | 83 | 144 | 83 | 134.1699982 | 250 | 186 | 72 | 266 |
| 11.20829964 | 9.116669655 | 28.75919914 | 861.75 | 28.29999924 | -3.400000095 | 31.70000076 | 19.75 | 0.733332992 | 21.91670036 | 0.733332992 | 426 | 54 | 24 | 23.17379951 | 135 | 79 | 130 | 79 | 66.62999725 | 250 | 176 | 77 | 267 |
| 10.73750019 | 9.891670227 | 32.32569885 | 777.7800293 | 26.79999924 | -3.799999952 | 30.60000038 | 14.63329983 | 2.033329964 | 20.14999962 | 1.116670012 | 552 | 63 | 36 | 16.77779961 | 167 | 113 | 152 | 121 | 355.7399902 | 244 | 192 | 62 | 689 |
| 11.4708004 | 9.891670227 | 32.22040176 | 782.7730103 | 27.60000038 | -3.099999905 | 30.70000076 | 15.38329983 | 2.733330011 | 20.9666996 | 1.783329964 | 531 | 58 | 35 | 15.51620007 | 158 | 109 | 140 | 117 | 98.69000244 | 235 | 168 | 70 | 418 |
| 11.39579964 | 10.20829964 | 32.71900177 | 789.7969971 | 27.89999962 | -3.299999952 | 31.20000076 | 15.2833004 | 2.616669893 | 21.01670074 | 1.666669965 | 531 | 59 | 35 | 15.57330036 | 157 | 109 | 143 | 117 | 138.6600037 | 246 | 143 | 71 | 376 |
| 11.00419998 | 9.041669846 | 28.43289948 | 861.7940063 | 28.10000038 | -3.700000048 | 31.79999924 | 19.56669998 | 0.449999988 | 21.66670036 | 0.449999988 | 440 | 54 | 24 | 22.42280006 | 138 | 81 | 132 | 81 | 97.98999786 | 249 | 88 | 76 | 320 |
| 11.44999981 | 9.183329582 | 28.8784008 | 856.9580078 | 28.5 | -3.299999952 | 31.79999924 | 19.88330078 | 1 | 22.06669998 | 1 | 429 | 53 | 24 | 22.38240051 | 133 | 80 | 129 | 80 | 62.36000061 | 244 | 77 | 77 | 346 |
| 11.19579983 | 9.091670036 | 28.68029976 | 855.3930054 | 28.20000076 | -3.5 | 31.70000076 | 19.63330078 | 0.766667008 | 21.79999924 | 0.766667008 | 434 | 54 | 24 | 22.73889923 | 136 | 80 | 131 | 80 | 87.12000275 | 247 | 43 | 77 | 327 |
| 11.19579983 | 9.091670036 | 28.68029976 | 855.3930054 | 28.20000076 | -3.5 | 31.70000076 | 19.63330078 | 0.766667008 | 21.79999924 | 0.766667008 | 434 | 54 | 24 | 22.73889923 | 136 | 80 | 131 | 80 | 96.5 | 249 | 46 | 77 | 337 |
| 11.32919979 | 9.091670036 | 28.68029976 | 856.0679932 | 28.29999924 | -3.400000095 | 31.70000076 | 19.75 | 0.883333027 | 21.93330002 | 0.883333027 | 431 | 53 | 24 | 22.32119942 | 134 | 80 | 129 | 80 | 59.45999908 | 246 | 164 | 77 | 357 |
| 11.32919979 | 9.091670036 | 28.68029976 | 856.0679932 | 28.29999924 | -3.400000095 | 31.70000076 | 19.75 | 0.883333027 | 21.93330002 | 0.883333027 | 431 | 53 | 24 | 22.32119942 | 134 | 80 | 129 | 80 | 34.99000168 | 250 | 79 | 77 | 321 |
| 11.32919979 | 9.091670036 | 28.68029976 | 856.0679932 | 28.29999924 | -3.400000095 | 31.70000076 | 19.75 | 0.883333027 | 21.93330002 | 0.883333027 | 431 | 53 | 24 | 22.32119942 | 134 | 80 | 129 | 80 | 43.56000137 | 246 | 8 | 77 | 321 |
| 11.1875 | 9.074999809 | 28.62779999 | 854.9539795 | 28.20000076 | -3.5 | 31.70000076 | 19.61669922 | 0.766667008 | 21.7833004 | 0.766667008 | 435 | 54 | 24 | 22.52260017 | 136 | 80 | 131 | 80 | 99 | 250 | 105 | 76 | 331 |
| 11.54170036 | 7.400000095 | 24.26230049 | 869.1339722 | 27.60000038 | -2.900000095 | 30.5 | 20.10000038 | 1.016669989 | 22.38330078 | 1.016669989 | 394 | 47 | 23 | 19.59889984 | 119 | 77 | 117 | 77 | 39.41999817 | 247 | 80 | 0 | 0 |
| 11.32919979 | 9.091670036 | 28.68029976 | 856.0679932 | 28.29999924 | -3.400000095 | 31.70000076 | 19.75 | 0.883333027 | 21.93330002 | 0.883333027 | 431 | 53 | 24 | 22.32119942 | 134 | 80 | 129 | 80 | 59.45999908 | 246 | 164 | 77 | 357 |
| 11.1875 | 9.074999809 | 28.62779999 | 854.9539795 | 28.20000076 | -3.5 | 31.70000076 | 19.61669922 | 0.766667008 | 21.7833004 | 0.766667008 | 435 | 54 | 24 | 22.52260017 | 136 | 80 | 131 | 80 | 99.16999817 | 249 | 140 | 76 | 299 |
| 11.4708004 | 9.891670227 | 32.22040176 | 782.7730103 | 27.60000038 | -3.099999905 | 30.70000076 | 15.38329983 | 2.733330011 | 20.9666996 | 1.783329964 | 531 | 58 | 35 | 15.51620007 | 158 | 109 | 140 | 117 | 54.68999863 | 250 | 139 | 71 | 417 |
| 10.92500019 | 9.949999809 | 32.41040039 | 779.6199951 | 27 | -3.700000048 | 30.70000076 | 14.83329964 | 2.216670036 | 20.38330078 | 1.283329964 | 547 | 61 | 36 | 15.68060017 | 163 | 112 | 149 | 121 | 320.7600098 | 236 | 101 | 68 | 546 |
| 11.54170036 | 7.400000095 | 24.26230049 | 869.1339722 | 27.60000038 | -2.900000095 | 30.5 | 20.10000038 | 1.016669989 | 22.38330078 | 1.016669989 | 394 | 47 | 23 | 19.59889984 | 119 | 77 | 117 | 77 | 29.36000061 | 201 | 25 | 76 | 547 |

**Table A4.** Pearson correlation matrix for the continuous environmental predictors.

| **Variable** | **BIO1** | **BIO2** | **BIO3** | **BIO4** | **BIO5** | **BIO6** | **BIO7** | **BIO8** | **BIO9** | **BIO10** | **BIO11** | **BIO12** | **BIO13** | **BIO14** | **BIO15** | **BIO16** | **BIO17** | **BIO18** | **BIO19** | **ELEVATION** | **SLOPE** | **ASPECT** | **PH** | **SOC** |
| --- | --- | --- | --- | --- | --- | --- | --- | --- | --- | --- | --- | --- | --- | --- | --- | --- | --- | --- | --- | --- | --- | --- | --- | --- |
| BIO1 | 1 | -0.1732 | -0.13306 | -0.05991 | 0.533504 | 0.928402 | -0.20302 | 0.049146 | 0.330146 | 0.594786 | 0.758216 | -0.11466 | -0.428 | 0.058797 | -0.39245 | -0.37467 | 2.36E-05 | -0.48814 | 0.019831 | -0.47464 | -0.29874 | -0.366 | -0.23302 | -0.23188 |
| BIO2 | -0.1732 | 1 | 0.972106 | -0.65591 | 0.073837 | -0.29663 | 0.283799 | -0.66023 | 0.364037 | -0.66514 | 0.270462 | 0.814983 | 0.904501 | 0.67883 | -0.28897 | 0.867049 | 0.690419 | 0.869663 | 0.682854 | 0.56429 | 0.539624 | 0.468368 | 0.576824 | 0.488612 |
| BIO3 | -0.13306 | 0.972106 | 1 | -0.81274 | -0.09696 | -0.18806 | 0.051078 | -0.78262 | 0.54469 | -0.7581 | 0.408005 | 0.909517 | 0.906564 | 0.812574 | -0.47744 | 0.910746 | 0.823805 | 0.884492 | 0.818038 | 0.622525 | 0.447282 | 0.456657 | 0.487469 | 0.567103 |
| BIO4 | -0.05991 | -0.65591 | -0.81274 | 1 | 0.453432 | -0.16991 | 0.531023 | 0.893645 | -0.85182 | 0.76395 | -0.69121 | -0.92151 | -0.68001 | -0.95261 | 0.845301 | -0.78527 | -0.95668 | -0.68819 | -0.95937 | -0.60788 | -0.06849 | -0.27283 | -0.11048 | -0.60008 |
| BIO5 | 0.533504 | 0.073837 | -0.09696 | 0.453432 | 1 | 0.259642 | 0.705698 | 0.423303 | -0.35407 | 0.683865 | 0.063794 | -0.34874 | -0.22966 | -0.37872 | 0.376133 | -0.3539 | -0.42173 | -0.31965 | -0.41834 | -0.48202 | 0.224863 | -0.16047 | 0.257634 | -0.35442 |
| BIO6 | 0.928402 | -0.29663 | -0.18806 | -0.16991 | 0.259642 | 1 | -0.50099 | -0.01664 | 0.494247 | 0.477849 | 0.792139 | -0.08654 | -0.48119 | 0.129423 | -0.53156 | -0.39085 | 0.07173 | -0.51251 | 0.088173 | -0.40143 | -0.37347 | -0.3726 | -0.32457 | -0.1186 |
| BIO7 | -0.20302 | 0.283799 | 0.051078 | 0.531023 | 0.705698 | -0.50099 | 1 | 0.39157 | -0.67993 | 0.262288 | -0.524 | -0.24905 | 0.147222 | -0.43436 | 0.727075 | -0.0304 | -0.43058 | 0.089548 | -0.43961 | -0.13746 | 0.475525 | 0.129558 | 0.469017 | -0.23061 |
| BIO8 | 0.049146 | -0.66023 | -0.78262 | 0.893645 | 0.423303 | -0.01664 | 0.39157 | 1 | -0.7462 | 0.769242 | -0.51758 | -0.93819 | -0.7417 | -0.93777 | 0.798459 | -0.85039 | -0.95374 | -0.76795 | -0.94631 | -0.68236 | -0.03158 | -0.49081 | -0.07051 | -0.50106 |
| BIO9 | 0.330146 | 0.364037 | 0.54469 | -0.85182 | -0.35407 | 0.494247 | -0.67993 | -0.7462 | 1 | -0.46252 | 0.787642 | 0.683303 | 0.298535 | 0.802543 | -0.91661 | 0.443099 | 0.789984 | 0.32801 | 0.788821 | 0.308265 | -0.05262 | 0.188133 | -0.02182 | 0.462315 |
| BIO10 | 0.594786 | -0.66514 | -0.7581 | 0.76395 | 0.683865 | 0.477849 | 0.262288 | 0.769242 | -0.46252 | 1 | -0.06248 | -0.83435 | -0.84864 | -0.74202 | 0.426115 | -0.89854 | -0.78334 | -0.89247 | -0.77229 | -0.79838 | -0.25237 | -0.48253 | -0.24521 | -0.62411 |
| BIO11 | 0.758216 | 0.270462 | 0.408005 | -0.69121 | 0.063794 | 0.792139 | -0.524 | -0.51758 | 0.787642 | -0.06248 | 1 | 0.492591 | 0.10844 | 0.643722 | -0.82542 | 0.213355 | 0.603648 | 0.06914 | 0.621404 | 0.050979 | -0.18633 | -0.127 | -0.11339 | 0.224612 |
| BIO12 | -0.11466 | 0.814983 | 0.909517 | -0.92151 | -0.34874 | -0.08654 | -0.24905 | -0.93819 | 0.683303 | -0.83435 | 0.492591 | 1 | 0.879846 | 0.966929 | -0.71502 | 0.9434 | 0.976229 | 0.886983 | 0.972142 | 0.730936 | 0.187082 | 0.444965 | 0.21566 | 0.592859 |
| BIO13 | -0.428 | 0.904501 | 0.906564 | -0.68001 | -0.22966 | -0.48119 | 0.147222 | -0.7417 | 0.298535 | -0.84864 | 0.10844 | 0.879846 | 1 | 0.74168 | -0.3042 | 0.973158 | 0.764796 | 0.990511 | 0.755863 | 0.786893 | 0.401716 | 0.522547 | 0.409792 | 0.553074 |
| BIO14 | 0.058797 | 0.67883 | 0.812574 | -0.95261 | -0.37872 | 0.129423 | -0.43436 | -0.93777 | 0.802543 | -0.74202 | 0.643722 | 0.966929 | 0.74168 | 1 | -0.85898 | 0.836664 | 0.996539 | 0.753795 | 0.996782 | 0.649557 | 0.040135 | 0.349666 | 0.070353 | 0.556648 |
| BIO15 | -0.39245 | -0.28897 | -0.47744 | 0.845301 | 0.376133 | -0.53156 | 0.727075 | 0.798459 | -0.91661 | 0.426115 | -0.82542 | -0.71502 | -0.3042 | -0.85898 | 1 | -0.45945 | -0.84187 | -0.33279 | -0.84745 | -0.36815 | 0.242598 | -0.11318 | 0.204933 | -0.36978 |
| BIO16 | -0.37467 | 0.867049 | 0.910746 | -0.78527 | -0.3539 | -0.39085 | -0.0304 | -0.85039 | 0.443099 | -0.89854 | 0.213355 | 0.9434 | 0.973158 | 0.836664 | -0.45945 | 1 | 0.862765 | 0.977232 | 0.855165 | 0.765044 | 0.301641 | 0.540675 | 0.317939 | 0.562286 |
| BIO17 | 2.36E-05 | 0.690419 | 0.823805 | -0.95668 | -0.42173 | 0.07173 | -0.43058 | -0.95374 | 0.789984 | -0.78334 | 0.603648 | 0.976229 | 0.764796 | 0.996539 | -0.84187 | 0.862765 | 1 | 0.781329 | 0.999084 | 0.676868 | 0.049982 | 0.37788 | 0.079048 | 0.566344 |
| BIO18 | -0.48814 | 0.869663 | 0.884492 | -0.68819 | -0.31965 | -0.51251 | 0.089548 | -0.76795 | 0.32801 | -0.89247 | 0.06914 | 0.886983 | 0.990511 | 0.753795 | -0.33279 | 0.977232 | 0.781329 | 1 | 0.769569 | 0.821082 | 0.379792 | 0.547897 | 0.382111 | 0.574876 |
| BIO19 | 0.019831 | 0.682854 | 0.818038 | -0.95937 | -0.41834 | 0.088173 | -0.43961 | -0.94631 | 0.788821 | -0.77229 | 0.621404 | 0.972142 | 0.755863 | 0.996782 | -0.84745 | 0.855165 | 0.999084 | 0.769569 | 1 | 0.666535 | 0.033304 | 0.354974 | 0.064732 | 0.556715 |
| ELEVATION | -0.47464 | 0.56429 | 0.622525 | -0.60788 | -0.48202 | -0.40143 | -0.13746 | -0.68236 | 0.308265 | -0.79838 | 0.050979 | 0.730936 | 0.786893 | 0.649557 | -0.36815 | 0.765044 | 0.676868 | 0.821082 | 0.666535 | 1 | 0.160362 | 0.399371 | 0.148354 | 0.61049 |
| SLOPE | -0.29874 | 0.539624 | 0.447282 | -0.06849 | 0.224863 | -0.37347 | 0.475525 | -0.03158 | -0.05262 | -0.25237 | -0.18633 | 0.187082 | 0.401716 | 0.040135 | 0.242598 | 0.301641 | 0.049982 | 0.379792 | 0.033304 | 0.160362 | 1 | 0.369458 | 0.434608 | 0.057407 |
| ASPECT | -0.366 | 0.468368 | 0.456657 | -0.27283 | -0.16047 | -0.3726 | 0.129558 | -0.49081 | 0.188133 | -0.48253 | -0.127 | 0.444965 | 0.522547 | 0.349666 | -0.11318 | 0.540675 | 0.37788 | 0.547897 | 0.354974 | 0.399371 | 0.369458 | 1 | 0.217122 | 0.249286 |
| PH | -0.23302 | 0.576824 | 0.487469 | -0.11048 | 0.257634 | -0.32457 | 0.469017 | -0.07051 | -0.02182 | -0.24521 | -0.11339 | 0.21566 | 0.409792 | 0.070353 | 0.204933 | 0.317939 | 0.079048 | 0.382111 | 0.064732 | 0.148354 | 0.434608 | 0.217122 | 1 | 0.597867 |
| SOC | -0.23188 | 0.488612 | 0.567103 | -0.60008 | -0.35442 | -0.1186 | -0.23061 | -0.50106 | 0.462315 | -0.62411 | 0.224612 | 0.592859 | 0.553074 | 0.556648 | -0.36978 | 0.562286 | 0.566344 | 0.574876 | 0.556715 | 0.61049 | 0.057407 | 0.249286 | 0.597867 | 1 |

**Figure A5**. Pearson correlation correlogram of the continuous environmental predictors used for variable selection.

**Model Calibration and evaluation**

The tables presented in this Appendix summarize the results of the MaxEnt model calibration procedure. Model calibration was performed in two sequential steps: (i) selection of the optimal number of cross-validation replicates and (ii) optimization of the regularization multiplier (RM), while all other model settings were maintained as described in the Materials and Methods section.

**Table A5.** Performance statistics obtained for different numbers of cross-validation replicates.

| **Number of replicates** | **Mean test AUC** | **SD of test AUC** | **Mean training AUC** | **Training–test AUC difference** | **Mean test gain** | **Regularized training gain** | **Test omission rate (10-percentile threshold)** | **Mean iterations** |
| --- | --- | --- | --- | --- | --- | --- | --- | --- |
| **10** | 0.9132 | 0.093 | 0.9438 | 0.0306 | 2.2179 | 2.1646 | 0.1500 | 222.0 |
| **9** | 0.9145 | 0.060 | 0.9428 | 0.0283 | 2.2221 | 2.1656 | 0.1481 | 226.7 |
| **8** | 0.9114 | 0.069 | 0.9431 | 0.0317 | 2.1116 | 2.1631 | 0.1979 | 232.5 |
| **7** | 0.9109 | 0.060 | 0.9429 | 0.0320 | 2.2298 | 2.1617 | 0.2024 | 222.9 |
| **6** | 0.9160 | 0.051 | 0.9424 | 0.0264 | 2.1630 | 2.1548 | 0.1917 | 236.7 |
| **5** | 0.9285 | 0.062 | 0.9445 | 0.0160 | 2.2156 | 2.1615 | 0.1400 | 200.0 |
| **4** | 0.9187 | 0.035 | 0.9432 | 0.0245 | 2.2173 | 2.1403 | 0.2381 | 225.0 |
| **3** | 0.9148 | 0.022 | 0.9448 | 0.0300 | 2.2827 | 2.1172 | 0.1528 | 220.0 |
| **2** | 0.9112 | 0.039 | 0.9498 | 0.0386 | 2.2656 | 1.8451 | 0.2308 | 170.0 |

**Table A6.** Performance statistics obtained for different values of the regularization multiplier.

| **Regularization multiplier** | **Mean test AUC** | **SD** | **Mean training AUC** | **Training–test AUC difference** | **Mean test gain** | **Regularized training gain** | **Test omission rate (10-percentile threshold)** | **Mean iterations** |
| --- | --- | --- | --- | --- | --- | --- | --- | --- |
| **0.25** | 0.9250 | 0.055 | 0.9523 | 0.0274 | 2.1076 | 2.5602 | 0.14 | 380 |
| **0.50** | 0.9302 | 0.053 | 0.9504 | 0.0202 | 2.1817 | 2.4127 | 0.14 | 256 |
| **0.75** | 0.9304 | 0.056 | 0.9478 | 0.0174 | 2.2079 | 2.2812 | 0.14 | 256 |
| **1.00** | 0.9285 | 0.062 | 0.9445 | 0.0161 | 2.2156 | 2.1615 | 0.14 | 200 |
| **1.25** | 0.9279 | 0.066 | 0.9419 | 0.0140 | 2.2226 | 2.0512 | 0.10 | 188 |
| **1.50** | 0.9261 | 0.071 | 0.9387 | 0.0125 | 2.2223 | 1.9485 | 0.10 | 160 |
| **1.75** | 0.9237 | 0.075 | 0.9351 | 0.0114 | 2.2141 | 1.8525 | 0.10 | 160 |
| **2.00** | 0.9224 | 0.079 | 0.9320 | 0.0096 | 2.1966 | 1.7627 | 0.10 | 144 |
