# Supplementary material for: Updated chorology, habitat ecology, conservation assessment and habitat suitability of Centaurea jankae in Romania and Bulgaria
Source: Front Plant Sci. 2026 Sep 18;17:1904573. doi: 10.3389/fpls.2026.1904573 (PMC13631827; doi:10.3389/fpls.2026.1904573)
Supplement: Supplementary file 1 [file SupplementaryFile1.zip › Supplementary Table S3.docx]

**Supplementary Table S3.** Descriptive summary of bioclimatic, topographic and edaphic variables associated with the five *Centaurea jankae* populations included in the environmental analyses in Romania and Bulgaria. Values are presented as mean ± SD, except for soil type. Country-level values are descriptive summaries only; no inferential statistical comparisons between Romania and Bulgaria were performed.

| **Variables** | **Unit** | **Romania** | **Bulgaria** |
| --- | --- | --- | --- |
| ***Bioclimatic variables*** | | | |
| Mean temperature – June | ℃ | 20.37 ± 0.31 | 19.21 ± 0.51 |
| Mean precipitation – June | mm | 51.39 ± 2.98 | 60.50 ± 2.59 |
| Annual Mean Temperature – BIO1 | ℃ | 11.22 ± 0.31 | 11.05 ± 0.43 |
| Temperature Seasonality – BIO4 | ℃ | 8.58 ± 0.08 | 7.79 ± 0.04 |
| Max Temperature of Warmest Month – BIO5 | ℃ | 27.80 ± 0.45 | 27.11 ± 0.51 |
| Min Temperature of Coldest Month – BIO6 | ℃ | -3.39 ± 0.38 | -3.49 ± 0.41 |
| Temperature Annual Range – BIO7 | ℃ | 31.19 ± 0.52 | 30.61 ± 0.10 |
| Mean Temperature of Warmest Quarter – BIO10 | ℃ | 21.86 ± 0.45 | 20.50 ± 0.48 |
| Annual Precipitation – BIO12 | mm | 421.64 ± 18.58 | 541.50 ± 10.90 |
| Precipitation of Driest Month – BIO14 | mm | 23.70 ± 0.47 | 35.50 ± 0.52 |
| Precipitation Seasonality – BIO15 | % | 21.50 ± 1.29 | 16.17 ± 0.68 |
| Precipitation of Driest Quarter – BIO17 | mm | 79.58 ± 1.90 | 111.00 ± 2.08 |
| Annual De Martonne aridity index – I_DMa_ | mm °C⁻¹ | 19.88 ± 1.10 | 25.73 ± 1.04 |
| June De Martonne aridity index – I_DMm_ | mm °C⁻¹ | 20.32 ± 1.32 | 24.87 ± 1.49 |
| ***Topographic variables*** | | | |
| Elevation | m a.s.l. | 67.2 ± 34.4 | 221.6 ± 124.8 |
| Aspect | ° | 162.2 ± 81.7 | 245.3 ± 56.6 |
| Slope | ° | 20.2 ± 17.6 | 16.5 ± 5.9 |
| ***Edaphic variables*** | | | |
| Organic carbon | % | 10.09 ± 1.8 | 7.2 ± 2.6 |
| Humus | % | 17.39 ± 3.1 | 12.4 ± 4.4 |
| Calcium (Ca) | mg/100 g soil | 5.53 ± 2.5 | 5.6 ± 0.7 |
| pH | – | 8.15 ± 0.2 | 8.4 ± 0.1 |
| Total phosphorus | mg/100 g soil | 1.27 ± 0.3 | 0.6 ± 0.2 |
| Ammonium nitrogen, N–NH₄⁺ | mg N/100 g soil | 0.003 ± 0.003 | 0.003 ± 0.001 |
| Nitrite nitrogen, N–NO₂⁻ | mg N/100 g soil | 0.002 ± 0.001 | 0.001 ± 0.001 |
| Nitrate nitrogen, N–NO₃⁻ | mg N/100 g soil | 0.15 ± 0.1 | 0.2 ± 0.1 |
| Organic nitrogen | mg N/100 g soil | 9.23 ± 2.5 | 6.6 ± 2.5 |
| Total nitrogen | mg N/100 g soil | 9.38 ± 2.5 | 6.8 ± 2.5 |
| Soil type | categorical | Calcic Kastanozems,  Calcic Chernozems | Calcic Chernozems |
