# Supplementary material for: Updated chorology, habitat ecology, conservation assessment and habitat suitability of Centaurea jankae in Romania and Bulgaria
Source: Front Plant Sci. 2026 Sep 18;17:1904573. doi: 10.3389/fpls.2026.1904573 (PMC13631827; doi:10.3389/fpls.2026.1904573)
Supplement: Supplementary file 1 [file SupplementaryFile1.zip › Supplementary Table S4.docx]

**Supplementary Table S4.** Historical and currently confirmed records of *Centaurea jankae* in Dobrogea, with associated geographic, ecological and field-verification information

| **Current locality** | **Historical locality / description** | **Plant association** | **EUNIS habitat** | **Elevation** **(m a.s.l.)** | **Slope** **(°)** | **Aspect** | **Soil / substrate** | **Country** | **References** | **Field status** |
| --- | --- | --- | --- | --- | --- | --- | --- | --- | --- | --- |
| Palazu Mic | Podișul Casimcei – via terasată a Palazului Mic | *Stipo ucrainicae–Festucetum valesiacae;* *Bombycilaeno erecti–Bothriochloetum ischaemi;* *Agropyro pontici–Thymetum zygioidis;*  *Festucetum callieri* | R1B Continental dry grassland (true steppe); R16 Perennial rocky grassland of Central and South-Eastern Europe | 45–100 | 3–33 | S, SSE, ENE, WSW | Calcic Chernozems | Constanța, Romania | Horeanu (1973); Dihoru and Pîrvu (1987); Negrean and Anastasiu (2002) | Confirmed |
| Dumbrăveni | Pădurea Dumbrăveni; Rezervația Dumbrăveni | NR | NR | NR | NR | NR | NR | Constanța, Romania | Parincu (1997); Parincu et al. (1998) | Not reconfirmed |
| Cotu Văii | Sudul Cotu Văii | NR | NR | NR | NR | NR | NR | Constanța, Romania | Negrean (2001), herbarium collection; Negrean and Anastasiu (2002) | Not reconfirmed |
| Cheile Dobrogei | | NR | NR | NR | NR | NR | NR | Constanța, Romania | Authors’ field observations (2017–2020) | Not reconfirmed |
| Caugagia | Între pădurea Babadag și satul Caugagia | NR | NR | NR | NR | NR | NR | Tulcea, Romania | Brândză (1884); Ghișa et al. (1964); Prodan (1913) | Not reconfirmed |
|  | Pe colinele aride dintre pădurea Babadag și Caugagia | NR | NR | NR | NR | NR | NR | Tulcea, Romania | Prodan (1911), herbarium collection | Not reconfirmed |
|  | Coasta aridă dintre Podișul Babadag și satul Caugagia | NR | NR | NR | NR | NR | NR | Tulcea, Romania | Prodan (1930) | Not reconfirmed |
|  | Între Caugagia și Tariverde | NR | NR | NR | NR | NR | NR | Tulcea, Romania | Prodan (1930, 1935); Ghișa et al. (1964) | Not reconfirmed |
| Jurilovca–Pădure | Pădurea de la Jurilovca | NR | NR | NR | NR | NR | NR | Tulcea, Romania | Prodan (1930) | Not reconfirmed |
| Jurilovca–Capul Doloșman | Capul Dolojman; Dealurile Cap-Dolojman | *Stipo ucrainicae–Festucetum valesiacae*  *Koelerio lobatae-Artemisietum lerchianae* | R1B Continental dry grassland (true steppe) | 21–30 | 40–54 | S, SSE, SSW, SW, WSW | Calcic Chernozems | Tulcea, Romania | Prodan (1930, 1939); Ghișa et al. (1964); Negrean (1978); Negrean and Constatinescu (1978, 1981), herbarium collection; Ciocârlan and Sârbu (1998); Negrean and Anastasiu (2002); Dihoru and Doniță (1970); Oprea (2005) | Confirmed |
| Codru | Pădurea Babadag, la intrarea șoselei dinspre Constanța | NR | NR | NR | NR | NR | NR | Tulcea, Romania | Dihoru and Doniță (1970); Dihoru and Pîrvu (1987) | Not reconfirmed |
|  | În antestepa de la sud de Babadag, pe coaste pietroase, sol scheletic stâncos | NR | NR | NR | NR | NR | Rocky skeletal soil (historical description) | Tulcea, Romania | Enculescu (1912), herbarium collection | Not reconfirmed |
|  | Jurilovca–Pădure | NR | NR | NR | NR | NR | NR | Tulcea, Romania | Prodan (1930) | Not reconfirmed |
| Slava Rusă – stone quarry | Cariera de la Slava Rusă | NR | NR | NR | NR | NR | NR | Tulcea, Romania | Dihoru (2009), herbarium collection | Not reconfirmed |
| Slava Rusă | | *Stipo ucrainicae–Festucetum valesiacae*  *Valerianello lasiocarpae–Chrysopogonetum grylli* | R1B Continental dry grassland (true steppe) | 105–109 | 1.3–14.3 | SE, SSE, ESE, ENE | Calcic Chernozems | Tulcea, Romania | Management Plan of the North Dobrogean Plateau | Confirmed |
| Fântâna Oilor | | NR | NR | NR | NR | NR | NR | Tulcea, Romania | Burduja and Horeanu (1976) | Not reconfirmed |
| Tariverde | Tariverde și pădurea Babadag | NR | NR | NR | NR | NR | NR | Tulcea, Romania | Dihoru and Doniță (1970) | Not reconfirmed |
| Nevsha | Taushan Tepe | *Stipo ucrainicae–Festucetum valesiacae* | R1B Continental dry grassland (true steppe) | 331–357 | 10–21 | W, NW | Calcic Chernozems | Varna, Bulgaria | Petrova (2007); Nikolov and Bancheva (2021) | Confirmed |
| Ravna | | *Stipo ucrainicae–Festucetum valesiacae;* *Valerianello lasiocarpae–Chrysopogonetum grylli* | R1B Continental dry grassland (true steppe); R1A Semi-dry perennial calcareous grassland (meadow steppe) | Mostly >100 | 5.9–21 | SW | Calcic Chernozems | Varna, Bulgaria | Petrova (2007) | Confirmed |
| Venchan | | NR | NR | 144–145 | 7.8 | WSW | NR | Varna, Bulgaria | Petrova (2007); Nikolov and Bancheva (2021) | Confirmed |

*NR, not reported in the original source; * no phytosociological relevé was available for this population.*
