# Supplementary material for: Updated chorology, habitat ecology, conservation assessment and habitat suitability of Centaurea jankae in Romania and Bulgaria
Source: Front Plant Sci. 2026 Sep 18;17:1904573. doi: 10.3389/fpls.2026.1904573 (PMC13631827; doi:10.3389/fpls.2026.1904573)
Supplement: Supplementary file 1 [file SupplementaryFile1.zip › Supplementary Table S5.docx]

**Supplementary Table S5.** Pairwise PERMANOVA comparisons of vegetation composition among the five investigated populations, based on Bray–Curtis dissimilarities. Adjusted p-values were calculated using the Holm correction.

| **Population_1** | **Population_2** | **pseudo_F** | **R2** | **p_raw** | **p_Holm** |
| --- | --- | --- | --- | --- | --- |
| Jurilovca | Palazu Mic | 17.43105 | 0.383681 | 1.00E-04 | 0.001 |
| Jurilovca | Slava Rusă | 26.09661 | 0.520926 | 1.00E-04 | 0.001 |
| Jurilovca | Nevsha | 18.12561 | 0.440738 | 1.00E-04 | 0.001 |
| Jurilovca | Ravna | 20.82292 | 0.475161 | 1.00E-04 | 0.001 |
| Palazu Mic | Slava Rusă | 8.013964 | 0.308064 | 1.00E-04 | 0.001 |
| Palazu Mic | Nevsha | 6.379951 | 0.272881 | 1.00E-04 | 0.001 |
| Palazu Mic | Ravna | 9.011725 | 0.346449 | 1.00E-04 | 0.001 |
| Slava Rusă | Nevsha | 11.71111 | 0.473921 | 2.00E-04 | 0.001 |
| Slava Rusă | Ravna | 16.49246 | 0.559209 | 2.00E-04 | 0.001 |
| Nevsha | Ravna | 3.647649 | 0.233112 | 0.0015 | 0.0015 |
